# Gut microbe–derived short-chain fatty acids regulate alphavirus arthritis and macrophage activation in mice

**DOI:** 10.1172/JCI202262

**Published:** 2026-07-15

**Authors:** Fang R. Zhao, Maksim Kleverov, Emma S. Winkler, Russell B. Williams, Hana Janova, Lindsay Droit, Leran Wang, Ting-ting Li, Leah Heath, Ana Jung, Matthias Mack, Megan T. Baldridge, Thaddeus S. Stappenbeck, Larissa B. Thackray, Chyi-Song Hsieh, Scott A. Handley, Chun-Jun Guo, Michael A. Fischbach, Maxim N. Artyomov, Michael S. Diamond

**Affiliations:** 1 Department of Medicine and; 2 Department of Pathology and Immunology, Washington University School of Medicine, St. Louis, Missouri, USA.; 3 Bioanalytical Chemistry Facility, Donald Danforth Plant Science Center, St. Louis, Missouri, USA.; 4 The Edison Family Center for Genome Sciences and Systems Biology, Washington University School of Medicine, St. Louis, Missouri, USA.; 5 Weill Cornell Medicine, Cornell University, New York, New York, USA.; 6 Department of Nephrology, University Hospital Regensburg, Regensburg, Germany.; 7 Department of Inflammation and Immunity, Cleveland Clinic, Cleveland, Ohio, USA.; 8 Department of Bioengineering and ChEM-H, Stanford University, Stanford, California, USA.; 9 Department of Molecular Microbiology, Washington University School of Medicine, St. Louis, Missouri, USA.

**Keywords:** Immunology, Infectious disease, Microbiology, Adaptive immunity, Innate immunity

## Abstract

Oral antibiotics can predispose to joint inflammation, but this phenomenon remains poorly understood. Here, we leverage mouse models of alphavirus-induced arthritis to investigate the roles of gut commensals, metabolites, and host immune mechanisms in promoting musculoskeletal inflammation. Mice treated with a short course of oral antibiotics exhibited worsened arthritis after chikungunya (CHIKV) or Mayaro virus infections. This phenotype was associated with loss of short-chain fatty acids (SCFAs), greater intestinal permeability, and activation of gut-associated immune cells and required TLR4 signaling, MyD88 expression, monocytes, antigen-specific and bystander CD4^+^ T cells, and proinflammatory cytokines. Administration of exogenous SCFAs or colonization of mice with bacterial species that generate SCFAs mitigated CHIKV-induced joint inflammation. scRNA-seq revealed that gut-derived SCFAs ameliorate the inflammatory phenotype of synovial CD4^+^ T cells, infiltrating monocytes, and resident osteoclast-like cells. Thus, antibiotic-triggered gut dysbiosis exacerbates alphavirus arthritis by shaping the inflammatory profile of both infiltrating and resident immune cells in joint tissues.

## Abstract 2

Zhao et al show that antibiotics-triggered changes in the microbiome exacerbate alphavirus arthritis by modulating inflammation by infiltrating and resident immune cells in joint tissues.

## Graphical abstract

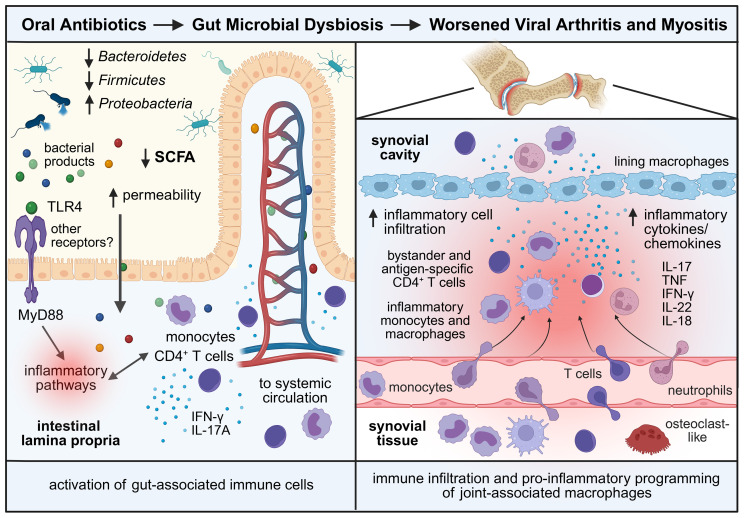

## Introduction

Antibiotic usage is linked to an increased risk for onset or relapse of inflammatory arthritis, including rheumatoid (RA) and juvenile idiopathic arthritis (1–6). Although a gut-joint linkage has been described (7–9), the basis for this increased risk with antibiotics use is not well understood, particularly how joint inflammation is regulated by interactions between gut microbes, mucosal barriers, and immune cells. Less is known about the effects of antibiotics and the commensal intestinal microbiota on inflammation in the context of pathogens that infect joint tissues.

Chikungunya virus (CHIKV) is a mosquito-transmitted alphavirus that causes epidemics of inflammatory arthritis. Recent resurgence in the Indian Ocean, Africa, and Asia and autochthonous cases in New York have triggered concern about another widespread epidemic. Three related alphaviruses (Mayaro [MAYV], Ross River, and O’nyong-nyong viruses) also cause musculoskeletal disease in the Americas, Oceania, and Africa, respectively (10). CHIKV infection causes an acute febrile syndrome in humans, with viremia, rash, myalgia, severe joint inflammation, and elevated systemic cytokines and chemokines. CHIKV arthritis clinically mimics RA, can persist for months to years after initial infection, and can cause bone erosions and joint damage (11–13).

In mice, subcutaneous inoculation of CHIKV leads to arthritis and myositis, like in humans (14–16). Inflammation is associated with tissue infiltration of monocytes, neutrophils, and T cells. However, there often is a discordance between viral burden and arthritis severity, suggesting that inflammation and pathology are not directly related to the viral burden in joint-associated tissues. Indeed, CHIKV infection of *Rag1^–/–^* mice lacking mature T and B cells leads to persistent local infection yet less joint swelling than infected WT mice (17). Mice lacking CD4^+^ T cells also have attenuated joint swelling after CHIKV infection without substantive effects on viral infection (18, 19). These data highlight the differential contributions of immune cells in restricting CHIKV infection and causing musculoskeletal disease.

Here, we use mouse models of alphavirus arthritis to investigate how gut commensals, their metabolites, and host immune responses promote joint inflammation in the setting of antibiotic-induced dysbiosis. Intestinal dysbiosis resulting from a short course of oral antibiotics results in increased gastrointestinal (GI) permeability due to loss of specific microbiota-derived short-chain fatty acids (SCFAs) and enhanced foot swelling and joint infiltration of monocytes and CD4^+^ T cells after CHIKV infection. The increased inflammation was dependent on gut-associated immune cells and required TLR4 and MyD88 signaling, with contributions from intestinal epithelial cells. The inflammatory phenotypes in antibiotic-treated mice were attenuated by neutralizing IL-17A, TNF, IL-18, or IFN-γ and not observed in alphavirus-infected germ-free (GF) mice. Reconstitution with SCFA-producing bacterial species or oral SCFA supplementation restored intestinal barrier integrity and reversed CHIKV-induced arthritis phenotypes in antibiotic-treated mice. Finally, SCFA supplementation modulated the inflammatory phenotypes of CD4^+^ T cells, monocytes, and osteoclast-like cells triggered by antibiotic treatment. Our data suggest that antibiotic-induced disruption of the intestinal microbiota results in loss of microbe-derived SCFAs, which drives inflammation in joint-associated tissues by shaping the functions of infiltrating and resident immune cells.

## Results

### Perturbation of the intestinal microbiota exacerbates CHIKV-induced arthritis.

**Figure 1:**
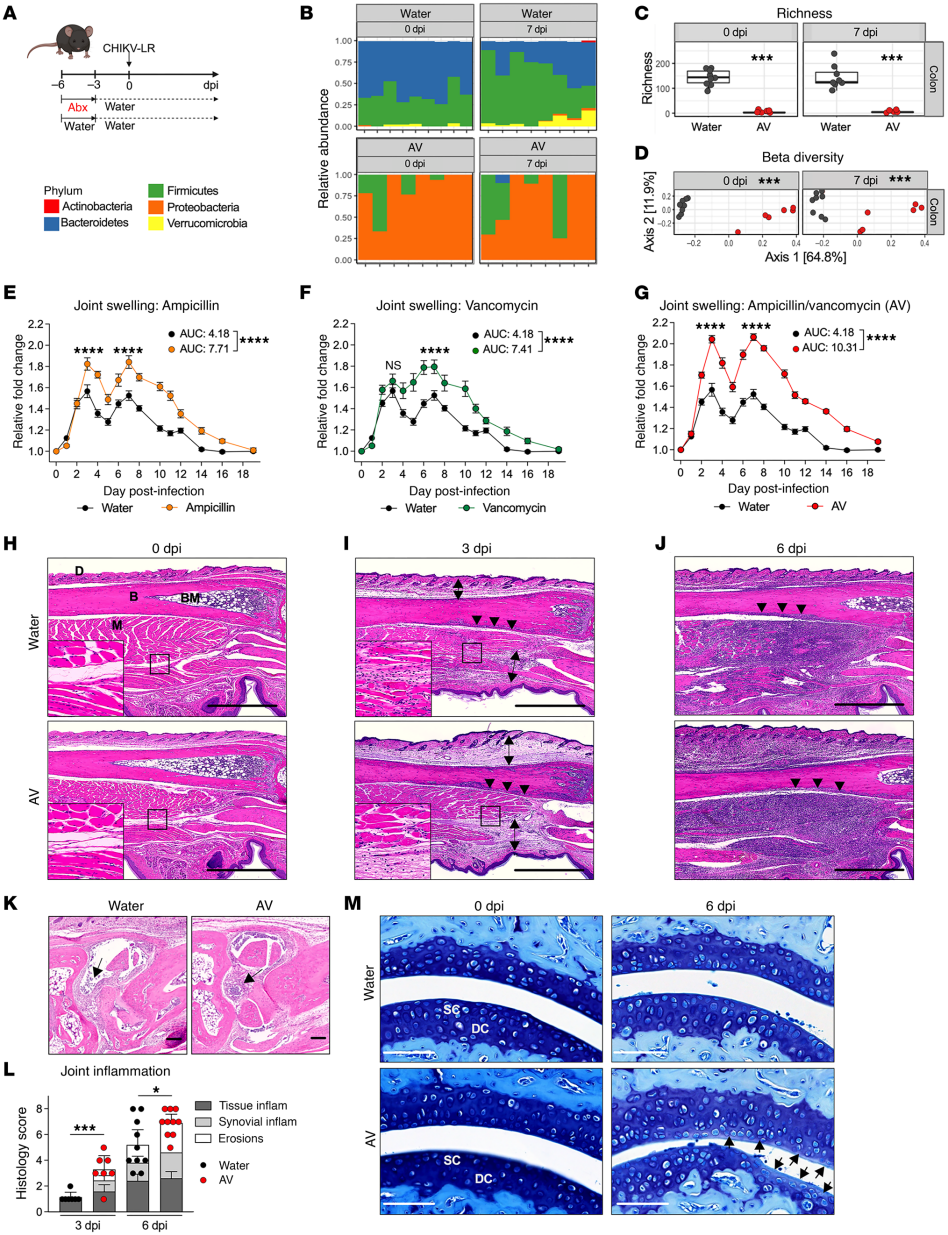
Depletion of the intestinal microbiota by oral antibiotics exacerbates musculoskeletal tissue inflammation after CHIKV infection. (**A**) Schematic of experimental setup. (**B**–**D**) Colonic contents were collected from water- or AV-treated C57BL/6J mice at 0 or 7 dpi. Relative abundance of bacterial phyla (**B**), number of bacterial taxa (richness) (**C**), and beta diversity (weighted UniFrac distance) (**D**) in colonic contents (2 experiments, *n* = 8–9 per group). (**E**–**G**) Foot swelling after CHIKV infection in mice treated with ampicillin (**E**, *n* = 10), vancomycin (**F**, *n* = 10), AV (**G**, *n* = 15), or water (**E**–**G**, *n* = 15). (**H**–**J**) H&E staining of foot tissues from water- or AV-treated mice at 0 (**H**), 3 (**I**), and 6 (**J**) dpi (*n* = 7–10 per group). Original magnification, ×2.5; scale bars: 1 mm. D, dermis; B, bone; BM, bone marrow; M, muscle. Double-headed arrows indicate edema. Arrowheads indicate periosteal inflammation. (**K**) H&E staining showing synovitis (arrows) in the mid-foot of water- or AV-treated mice at 6 dpi. Original magnification, ×5; scale bars: 100 μm. (**L**) Scoring of joint inflammation and tissue damage. (**M**) Toluidine blue staining of foot tissues from water- or AV-treated mice at 0 and 6 dpi (representative of *n* = 2 uninfected and *n* = 8 infected mice per group). Original magnification, ×40; scale bars: 100 μm. SC, superficial noncalcified cartilage; DC, deeper calcified cartilage. Arrowheads indicate destaining (proteoglycan loss) of superficial hyaline cartilage. Statistical analysis: **C**, Wilcoxon’s test; **D**, permutational multivariate ANOVA (Adonis); **E**–**G**, mean ± SEM, 2-way ANOVA with Šidák’s post test or 1-way ANOVA with Dunnett’s post test for AUC; **L**, unpaired 2-tailed *t* test. ****P < 0.0001; ***P < 0.001; *P < 0.05.

To assess the role of the microbiota on disease severity, we treated C57BL6/J mice with a short, 3-day course of minimally absorbable oral antibiotics, ampicillin, and vancomycin (Figure 1A). This treatment, which was chosen to limit systemic exposure, alters the bacterial community structure in the GI tract by depleting bacteria primarily from the *Bacteroidetes* phylum, leading to overrepresentation of *Proteobacteria* and *Firmicutes* (Figure 1, B–D, and Supplemental Figure 1, A–C; supplemental material available online with this article; https://doi.org/10.1172/JCI202262DS1). While treatment with either vancomycin or ampicillin alone increased CHIKV-induced foot swelling, the 2 antibiotics together resulted in a more pronounced phenotype (Figure 1, E–G); consequently, we used the ampicillin and vancomycin (AV) combination for the remainder of our studies. Notably, the enhanced inflammatory phenotype persisted for at least 8 weeks after antibiotic cessation (Supplemental Figure 1, D–G). Similar increases in joint swelling were also observed in AV-treated mice after infection with MAYV, a related arthritogenic alphavirus (Supplemental Figure 1H).

Histological examination of ipsilateral foot tissues at 3 days postinfection (dpi) showed greater soft tissue edema in AV-treated CHIKV-infected mice than water-treated, infected, or uninfected controls (Figure 1, H and I). Mononuclear cell infiltration into the joint and adjacent muscle was seen in both water- and AV-treated CHIKV-infected mice at this time point (Figure 1I). By 6 dpi, extensive soft tissue inflammation, myositis with muscle degeneration and necrosis, and periostitis were evident in tissues from all CHIKV-infected mice (Figure 1J). However, synovitis and erosion of synovial membranes were more severe in AV-treated mice (Figure 1, K and L).

**Figure 2:**
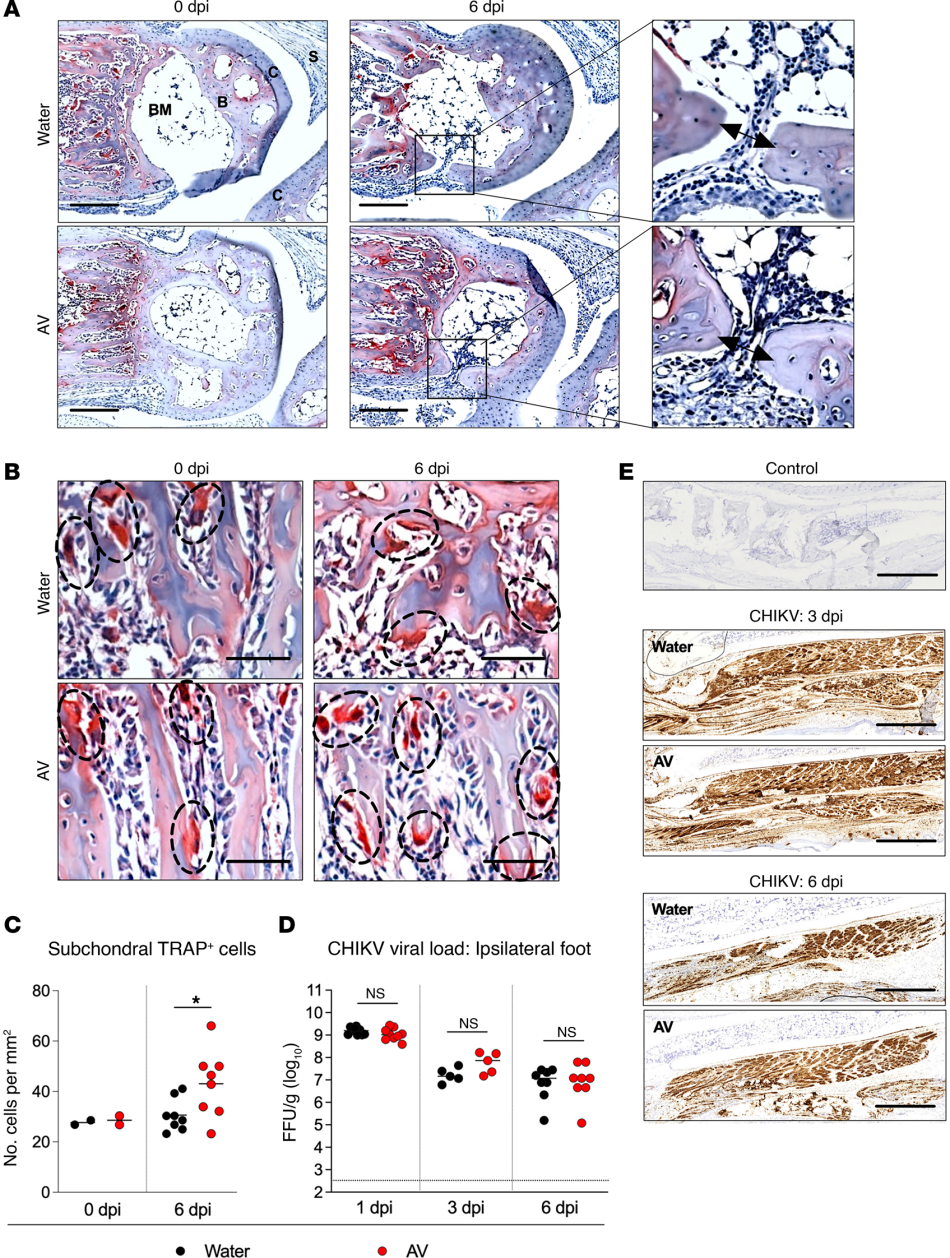
Oral antibiotics increase subchondral osteoclasts without altering viral infection during CHIKV infection. (**A** and **B**) TRAP staining of foot tissues from water- or AV-treated mice at 0 and 6 dpi (representative of *n* = 8 per group); red staining shows TRAP^+^ cells. Original magnification, ×10; scale bars: 200 μm. B, bone; BM, bone marrow; S, synovium; C, cartilage. In the higher magnification views, double-headed arrows indicate the width of the vascular channels. Dashed ovals in **B** encircle subchondral TRAP^+^ osteoclasts (red, nucleated). (**C**) Quantitation of subchondral TRAP^+^ osteoclasts in water- and AV-treated mice at 0 and 6 dpi. (**D**) CHIKV infection in ipsilateral feet of water- and AV-treated mice at 1, 3, and 6 dpi was determined by focus-forming assay (2 experiments, *n* = 5–8 per group). FFU, focus-forming units. (**E**) CHIKV RNA in situ hybridization of foot tissue sections from Zika virus–infected mice (negative control, top panel) and CHIKV-infected, water- or AV-treated mice at 3 dpi (middle panels) or 6 dpi (bottom panels) (representative of *n* = 3 per group). Original magnification, ×2.5; scale bars: 1 mm. Statistical analysis: **C** and **D**, Mann-Whitney test. *P < 0.05.

Given the association between inflammation and cartilage damage, we evaluated articular cartilage integrity using toluidine blue staining. At 6 dpi, AV-treated mice showed reduced proteoglycan content in the superficial noncalcified cartilage layer compared with water-treated controls (Figure 1M). We also performed tartrate resistant acid phosphatase (TRAP) staining to visualize osteoclasts. In both water- and AV-treated CHIKV-infected mice, we observed transcortical vascular channels close to the epiphysis and subchondral bone, structures that expand during inflammation and lead to osteoclast-induced bone erosions (20–22), with increased cellularity in AV-treated mice (Figure 2A). AV treatment was also associated with increased density of subchondral TRAP^+^ osteoclasts at 6 dpi (Figure 2, A–C).

To determine whether antibiotic-mediated exacerbation of CHIKV arthritis was related to increased viral burden, we quantified viral load and visualized viral RNA within joint tissues using in situ hybridization. Notably, viral titers in the ipsilateral foot were comparable between water- and AV-treated mice at both 3 and 6 dpi, and intense viral RNA staining was detected in the periosteum, articular cartilage, synovium, and muscles in both groups (Figure 2, D and E). These data suggest that enhanced musculoskeletal inflammation in AV-treated mice is not due to increased viral replication or altered viral distribution in joint tissues.

### Oral antibiotic treatment increases immune cell infiltration and cytokine levels in musculoskeletal tissues after CHIKV infection.

**Figure 3:**
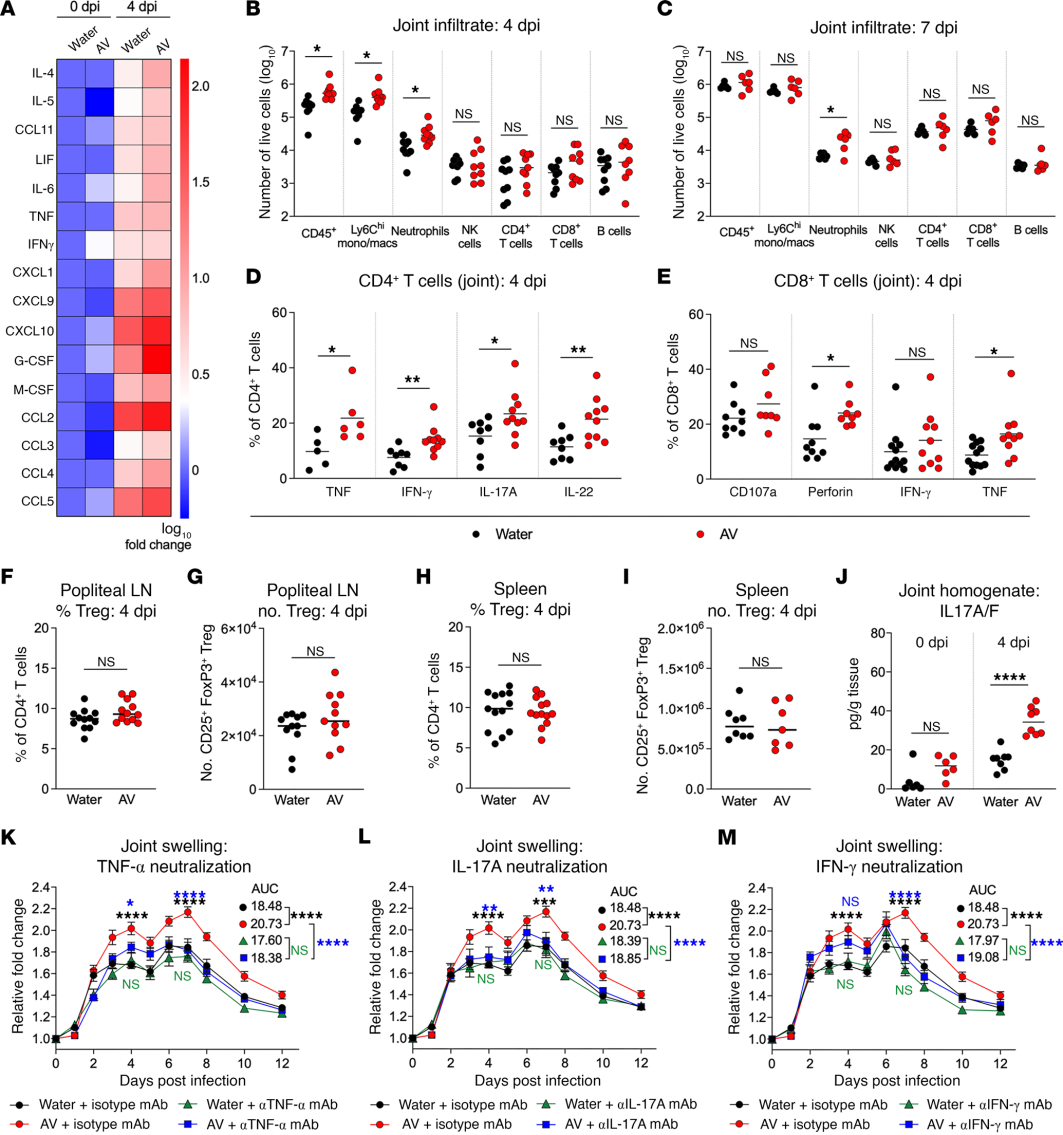
Oral antibiotics increase joint inflammation during CHIKV infection. (**A**) Heatmap of cytokine and chemokine levels in foot-associated musculoskeletal tissue homogenates from water- or AV-treated mice at 0 and 4 dpi (2 experiments, *n* = 5–8 per group, log_10_ fold differences in protein levels are relative to water-treated animals at 0 dpi; see also Supplemental Figure 1J and Supplemental Tables 1 and 2). (**B** and **C**) Numbers of immune cells in joint-associated tissues in the ipsilateral feet of water- or AV-treated mice at 4 (**B**) or 7 (**C**) dpi (2 experiments, *n* = 6–9 per group). (**D**) Percentages of IFN-γ–, TNF-, IL-17–, and IL-22–producing CD4^+^ T cells in the ipsilateral feet of water- and AV-treated mice at 4 dpi (2–3 experiments, *n* = 6–11 per group). (**E**) Percentages of IFN-γ– and TNF-producing, perforin-expressing, or CD107a-cycling CD8^+^ T cells in the ipsilateral feet of control and AV-treated mice at 4 dpi (3 experiments, *n* = 8–17 per group). (**F**–**I**) Percentages and numbers CD4^+^CD25^+^FoxP3^+^ Tregs in the draining popliteal lymph nodes (**F** and **G**) and spleens (**H** and **I**) of water- and AV-treated mice at 4 dpi. (**J**) IL-17A/F levels in joint-associated tissue homogenates from ipsilateral feet (2 experiments, *n* = 5–8 per group). (**K**–**M**) Foot swelling of water- and AV-treated mice that were administered isotype control or neutralizing mAb against TNF (**K**), IL-17A (**L**), or IFN-γ (**M**) (2 experiments, *n* = 8–9 per group). Statistical analysis: **B**–**J**, unpaired *t* test; **K**–**M**, 2-way ANOVA with Šidák’s post test or 1-way ANOVA with Dunnett’s post test for AUC; mean values ± SEM are shown. ****P < 0.0001; ***P < 0.001; **P < 0.01; *P < 0.05.

We next evaluated the effects of AV treatment on inflammation in CHIKV-infected joint-associated tissues. At 4 dpi, AV-treated mice had higher levels of IL-4, IL-5, CCL11, LIF, IL-6, IFN-γ, TNF, M-CSF, and G-CSF, as well as the IFN-induced chemokines, CCL2, CCL3, CCL4, and CXCL10, in the ipsilateral foot (Figure 3A and Supplemental Figure 1, I and J) compared with water-treated controls. To characterize immune cell infiltrates, we performed flow cytometry analyses of cell composition in the ipsilateral foot after CHIKV infection (Supplemental Figure 2A). AV-treated mice exhibited increased numbers of monocytes and neutrophils at 4 dpi and increased neutrophils at 7 dpi (Figure 3, B and C). No significant differences were observed in numbers of NK, T, or B cell populations (Figure 3, B and C).

We also assessed whether AV treatment altered qualitative T cell responses in joint-associated tissues after CHIKV infection. At 4 dpi, AV-treated mice showed increased proportions and numbers of TNF-, IFN-γ–, IL-17A–, and IL-22–producing CD4^+^ T cells in the joint (Figure 3D and Supplemental Figure 2B). In contrast, CD8^+^ T cells displayed similar percentages of degranulation and IFN-γ production in both groups, although AV-treated mice showed a small increase in the proportion of perforin- and TNF-expressing CD8^+^ T cells (Figure 3E). Significant differences in the percentages or numbers of FoxP3^+^ Tregs were not detected between water- and AV-treated mice in the draining popliteal lymph node (Figure 3, F and G) or spleen (Figure 3, H and I) at 4 dpi.

Using enzyme-linked immunosorbent assays, we confirmed that AV-treated mice had elevated levels of IL-17A, TNF, and IFN-γ in joint-associated tissues at 4 dpi compared with water-treated mice (Figure 3J and Supplemental Figure 1I). To determine their functional contribution, we administered neutralizing mAbs to water- and AV-treated mice. Treatment with anti-TNF or anti–IL-17A mAb improved foot swelling in antibiotic-treated mice following CHIKV infection (Figure 3, K and L). Neutralization of IFN-γ in AV-treated mice led to comparatively smaller reductions in inflammation at 3–4 dpi but a marked reduction in swelling at and after 7 dpi (Figure 3M). Thus, antibiotic-mediated dysbiosis promotes polarization of CD4^+^ T cells and infiltration of myeloid cells into the joint, resulting in higher levels of cytokines that augment foot swelling after CHIKV infection.

### Anaerobic commensal bacteria regulate musculoskeletal inflammation after CHIKV infection.

**Figure 4:**
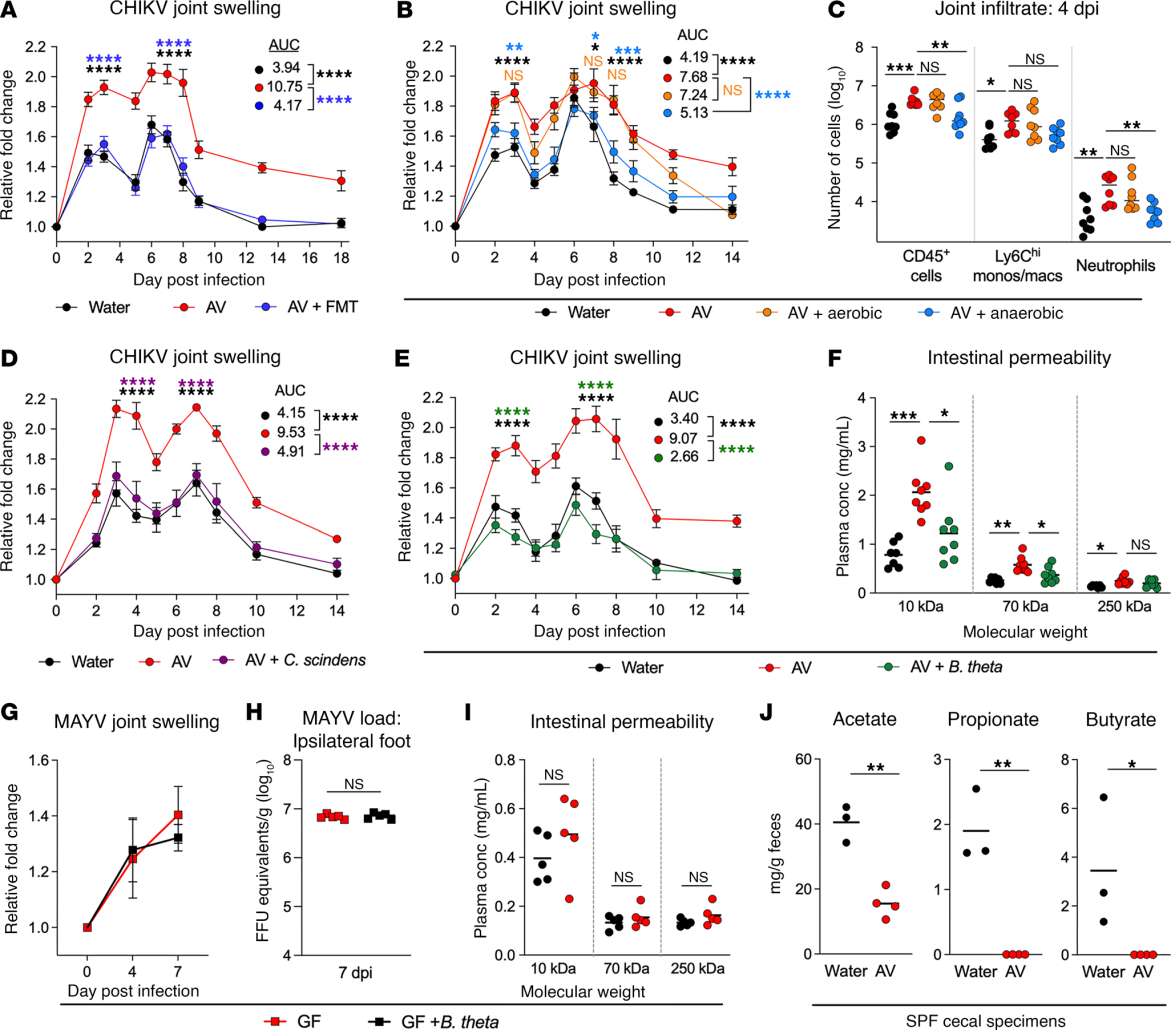
Microbial dysbiosis results in reduced SCFA production, increased intestinal permeability, and enhanced joint swelling. (**A**) Foot swelling after CHIKV infection in water-treated, AV-treated, or AV-treated mice after FMT (AV + FMT) (3 experiments, *n* = 13–14 per group). (**B** and **C**) Foot swelling (**B**) and immune cell infiltrates in the feet (**C**) after CHIKV infection of water-treated, AV-treated, or AV-treated mice colonized with either aerobic or anaerobic fecal cultures (2 experiments, *n* = 8–9 per group). (**D** and **E**) Foot swelling after CHIKV infection of water-treated, AV-treated, or AV-treated mice colonized with either *C*. *scindens* (**D**) or *B*. *thetaiotaomicron* (**E**) (2 experiments, *n* = 9 per group). (**F**) Plasma concentrations of 10, 70, and 250 kDa dextrans at 1.5 h after oral gavage (2 experiments, *n* = 8). (**G** and **H**) GF mice received PBS or colonization with *B*. *thetaiotaomicron* via oral gavage prior to MAYV infection. Foot swelling after MAYV infection (**G**) and MAYV burden (**H**) in tissue homogenates from the feet of GF mice or GF mice colonized with *B*. *thetaiotaomicron*. (**I**) Plasma concentrations of 10, 70, and 250 kDa dextrans at 1.5 h after oral gavage. (**J**) Fecal SCFA levels in water- or AV-treated mice. Statistical analysis: **A**, **B**, **D**, and **E**, 2-way ANOVA with Dunnett’s post test; AUC analyses were performed with 1-way ANOVA with Šidák’s post test; mean values ± SEM. **C** and **F**, 1-way ANOVA with Šidák’s post test. **H**, Mann-Whitney test. **I** and **J**, unpaired *t* test. ****P < 0.0001; ***P < 0.001; **P < 0.01; *P < 0.05.

We next evaluated whether reconstitution with selected bacterial species could rescue the exacerbated joint swelling induced by AV treatment. Fecal microbial transplant (FMT) from untreated mice reversed the increased CHIKV-induced joint swelling observed in AV-treated mice (Figure 4A and Supplemental Figure 2C). To determine whether specific communities modulated the severity of musculoskeletal inflammation after CHIKV infection, we colonized AV-treated mice with anaerobic or aerobic cultures derived from FMT. Reconstitution with anaerobic, but not aerobic, cultures reversed CHIKV-induced tissue swelling and immune cell infiltration to levels seen in untreated, infected mice (Figure 4, B and C).

We next tested whether individual anaerobic bacterial species could modulate musculoskeletal inflammation after CHIKV infection. We focused on *Bacteroides* and *Clostridium* species since these commensals are depleted by the AV regimen (Figure 1B and Supplemental Figure 1A) and are known to influence host immunity (23, 24). Remarkably, colonization of AV-treated mice with either *Clostridium scindens* or *Bacteroides thetaiotaomicron* reversed CHIKV-induced musculoskeletal inflammation to levels comparable with water-treated animals (Figure 4, D and E). These data demonstrate functional redundancy in the ability of different commensal gut bacteria to regulate virus-induced joint inflammation.

### Antibiotic treatment increases paracellular intestinal permeability.

The gut microbiota interfaces with the intestinal epithelium to modulate permeability through transcellular (transcytosis or carrier-dependent transport) and paracellular (tight junction–dependent) routes; accordingly, gut dysbiosis can promote translocation of microbes, microbial products, or metabolites (25). To evaluate the effect of AV treatment on intestinal permeability, we measured translocation of orally gavaged fluorescently conjugated dextrans of different molecular weights from the intestinal lumen into the peripheral blood. We observed increased plasma levels of 10, 70, and 250 kDa dextrans in AV-treated mice compared with water-treated animals (Figure 4F), although the larger 250 kDa dextrans were minimally translocated in both groups. Colonization of AV-treated mice with *B*. *thetaiotaomicron* restored intestinal barrier integrity (Figure 4F), linking microbial composition to intestinal permeability and CHIKV-induced inflammation.

We explored the requisite role of intestinal microbes in exacerbating alphavirus-induced musculoskeletal inflammation. GF mice that were monocolonized with *B*. *thetaiotaomicron* or maintained as GF were infected with the related alphavirus, MAYV, and evaluated for foot swelling; MAYV was used, instead of CHIKV, because our gnotobiotic facility operates under A-BSL2 conditions (CHIKV requires A-BSL3 containment). Noncolonized and *B*. *thetaiotaomicron*–colonized GF mice exhibited similar levels of foot swelling at 4 and 7 days after MAYV infection (Figure 4G), with similar levels of viral RNA in the foot (Figure 4H). Dextran translocation via paracellular intestinal permeability was also similar between GF mice and those monocolonized with *B*. *thetaiotaomicron* (Figure 4I). Together, these data suggest that alteration in specific gut microbial communities, rather than depletion or an absence of microbiota, regulate intestinal permeability and joint inflammation during alphavirus infection.

### SCFAs regulate intestinal permeability and joint inflammation after CHIKV infection.

The intestinal microbiota can regulate immune responses through direct effects on immune cells or by indirect modulation of the gut epithelial barrier (26). We hypothesized that exacerbated CHIKV-induced foot swelling after AV treatment might arise from loss of specific commensal bacteria and their metabolites. *Bacteroidetes*, the major phylum of bacteria depleted by our antibiotic regimen (Figure 1B and Supplemental Figure 1A), and *Clostridia* are primary producers of SCFAs and biotransformers of secondary bile acids (BAs) (27, 28). Indeed, we previously found that levels of deoxycholic acid (DCA), a secondary BA, are reduced in antibiotic-treated mice (29). However, oral DCA supplementation worsened intestinal permeability and foot swelling after CHIKV infection (Supplemental Figure 2, D–F), consistent with reports that secondary BAs disrupt intestinal barrier functions (30, 31).

**Figure 5:**
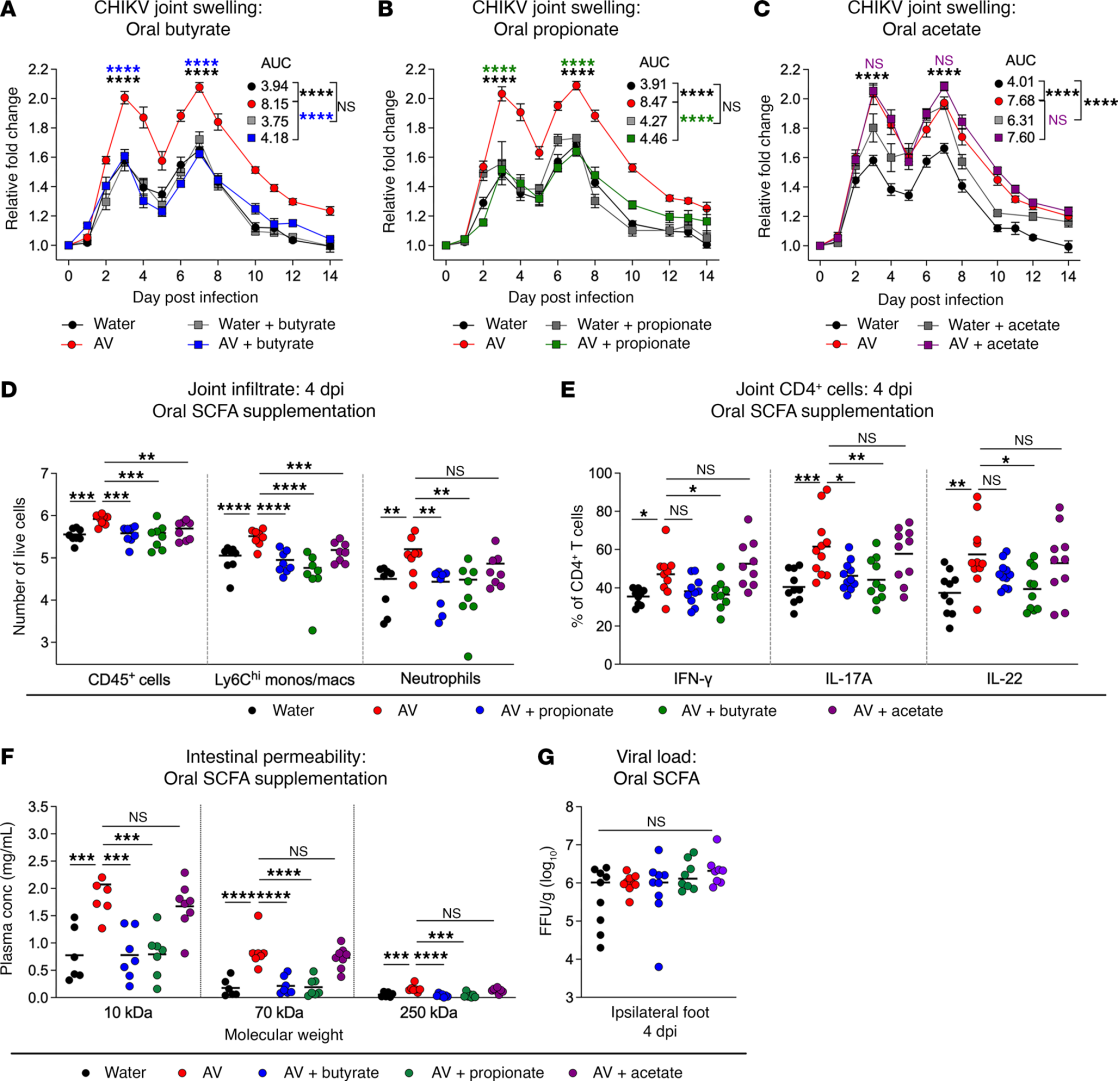
Oral supplementation with exogenous SCFA rescues antibiotic-mediated exacerbation of CHIKV-induced foot swelling. Mice were treated with water or AV, with or without SCFA supplementation, and subsequently inoculated with CHIKV. (**A**–**C**) Foot swelling after CHIKV infection in water- or AV-treated mice with or without butyrate (**A**), propionate (**B**), or acetate (**C**) supplementation (2–3 experiments, *n* = 10–12 per group). (**D**) Numbers of joint-associated immune cells at 4 dpi (2 experiments, *n* = 8 per group). (**E**) Percentages of cytokine-producing CD4^+^ T cells at 4 dpi (3 experiments, *n* = 8–11 per group). (**F**) Plasma concentrations of 10, 70, and 250 kDa dextran at 1.5 h after oral gavage (2 experiments, *n* = 6–7 per group). (**G**) CHIKV infection at 4 dpi in tissue homogenates from the ipsilateral feet (2 experiments, *n* = 8–9 per group). Statistical analysis: **A**–**C**, 2-way ANOVA with Dunnett’s post test; AUC analyses were performed with 1-way ANOVA with Šidák’s post test; mean values ± SEM. **D**–**G**, 1-way ANOVA with Šidák’s post test. ****P < 0.0001; ***P < 0.001; **P < 0.01; *P < 0.05.

The 3 most abundant SCFAs (acetate, propionate, and butyrate) were reduced after AV treatment (Figure 4J and Supplemental Figure 3, A and B). These SCFAs promote epithelial homeostasis and barrier function (32), as well as regulatory immune responses (33–35). Thus, we evaluated whether oral supplementation with these individual SCFAs could restore intestinal barrier integrity and modulate CHIKV-induced foot swelling in AV-treated mice (Supplemental Figure 2D). Orally delivered butyrate or propionate attenuated foot swelling of AV-treated mice to levels seen in water-treated mice (Figure 5, A–C). Butyrate or propionate treatment also reduced the numbers of infiltrating monocytes and neutrophils; decreased the proportions of CD4^+^ T cells producing IFN-γ, IL-17, and IL-22; and restored intestinal barrier integrity (Figure 5, D–F). In contrast, acetate had no effect on these phenotypes (Figure 5, C–F). The protective effects of butyrate or propionate were independent of viral burden (Figure 5G) and observed only in AV-treated mice; SCFA supplementation in conventionally housed, water-treated mice did not affect foot swelling caused by CHIKV infection (Figure 5, A–C), in contrast to results from a prior study (36). Together, these data identify specific SCFAs as key mediators linking the gut microbiota to intestinal barrier integrity and virus-induced joint inflammation.

### Rescue of enhanced joint inflammation by bacteria requires microbe-derived SCFAs.

**Figure 6:**
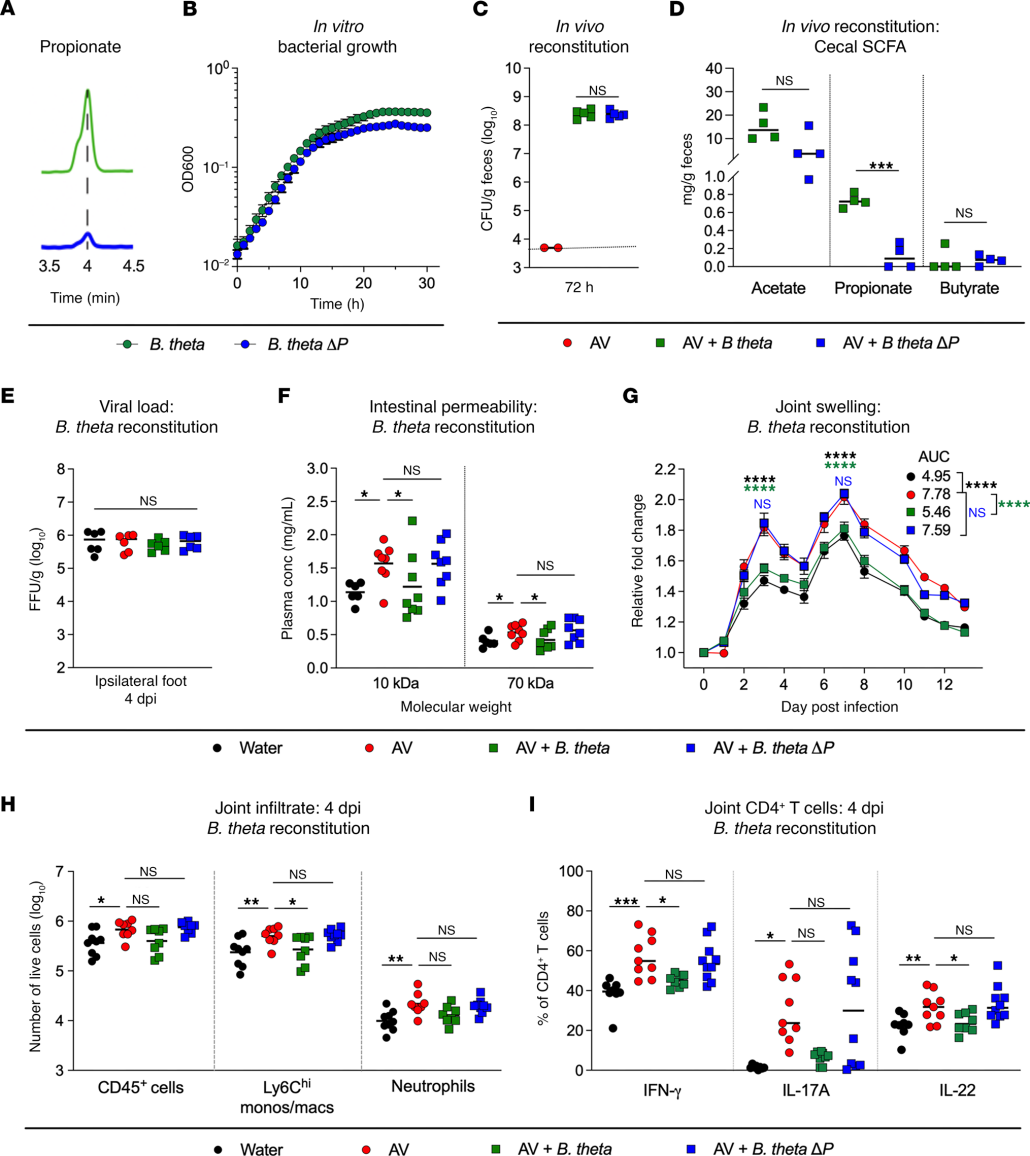
Rescue of CHIKV-induced foot swelling by bacterial colonization depends on microbe-derived SCFA. (**A**) In vitro production of propionate by isogenic WT and mutant (Δ*P*) *B*. *thetaiotaomicron*. (**B**) In vitro growth properties of isogenic WT and mutant *B*. *thetaiotaomicron*. (**C** and **D**) AV-treated mice were gavaged with sterile PBS or with WT or mutant *B*. *thetaiotaomicron*. (**C**) Fecal colony-forming unit counts on *Bacteroides* bile esculin agar. (**D**) Cecal SCFA levels determined by gas chromatography–mass spectrometry. (**E**–**I**) Water-treated, AV-treated, or AV-treated mice colonized with either WT or mutant *B*. *thetaiotaomicr*o*n* were inoculated with CHIKV. (**E**) CHIKV infection at 4 dpi in homogenates of the ipsilateral feet (2 experiments, *n* = 6 per group). (**F**) Plasma concentrations of 10 and 70 kDa dextran were measured at 1.5 h after gavage (3 experiments, *n* = 10–13 per group). (**G**) Foot swelling after CHIKV infection (2 experiments, *n* = 10 per group). Numbers of joint-associated immune cells (**H**) and percentages of cytokine-producing CD4^+^ T cells at 4 dpi (**I**) (2 experiments, *n* = 8–10 per group). Statistical analysis: **C** and **E**, Mann-Whitney test; **D**, unpaired *t* test; **F**, **H**, and **I**, 1-way ANOVA with Šidák’s post test. **G**, 2-way ANOVA with Tukey’s post test; AUC was analyzed using 1-way ANOVA with Dunnett’s post test; mean values ± SEM. ****P < 0.0001; ***P < 0.001; **P < 0.01; *P < 0.05.

To link our findings on SCFAs and bacterial reconstitution, we generated an isogenic clone of *B*. *thetaiotaomicron* deficient in propionate synthesis by targeting methylmalonyl-CoA mutase (*ΔBT2090-2091*) to assess whether the protective effect of *B*. *thetaiotaomicron* depends on its ability to produce SCFA. Loss of propionate production in *ΔBT2090-2091* *B*. *thetaiotaomicron* (*B*. *theta* *delta P*) was validated by gas chromatography–mass spectrometry on extracts from in vitro cultures (Figure 6A).

WT and *ΔBT2090-2091* *B*. *thetaiotaomicron* showed similar growth kinetics in vitro and after colonization in AV-treated mice (Figure 6, B and C). We confirmed that cecal samples from mice colonized with *ΔBT2090-2091* *B*. *thetaiotaomicron* had less propionate than animals colonized with WT *B*. *thetaiotaomicron* (Figure 6D and Supplemental Figure 3, A and B). In contrast to the parental strain, colonization with the *ΔBT2090-2091* *B*. *thetaiotaomicron* failed to restore intestinal barrier integrity, diminish monocyte infiltrates or inflammatory cytokine production by CD4^+^ T cells in the joint, or reduce CHIKV-induced foot swelling (Figure 6, E–I).

### Enhanced joint inflammation after oral antibiotic treatment requires MyD88 signaling.

**Figure 7:**
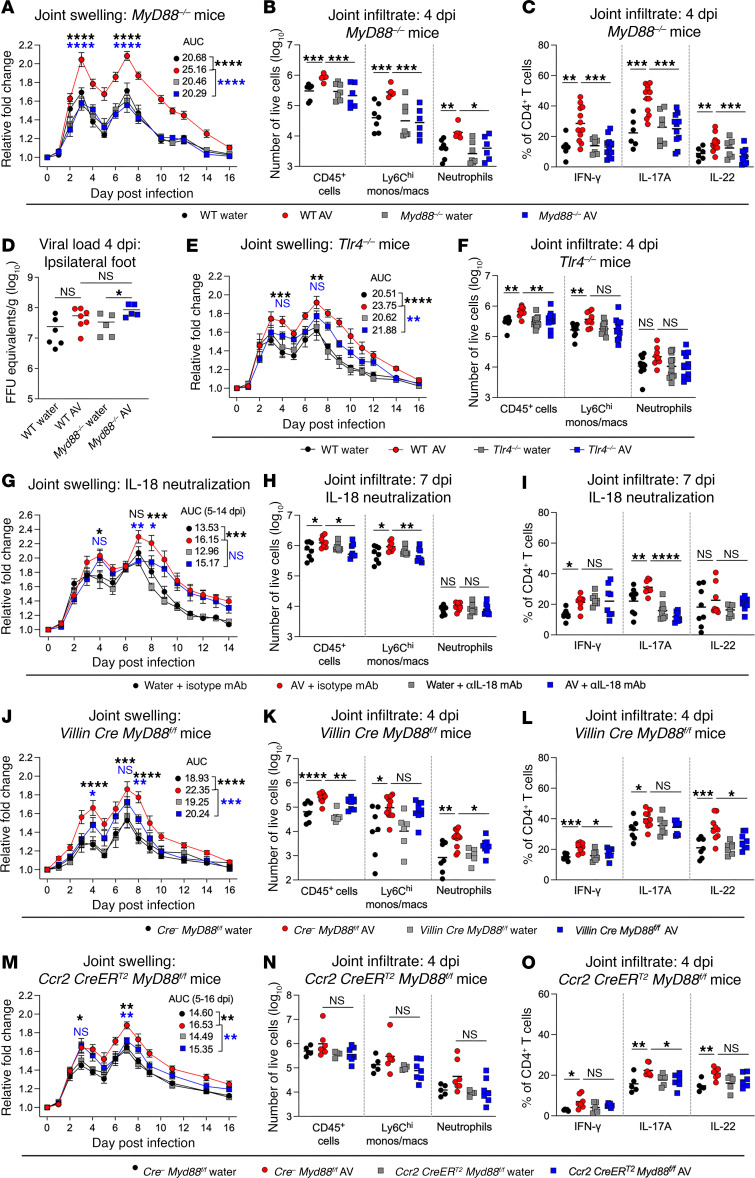
MyD88 signaling in intestinal epithelial cells promotes CHIKV-induced joint inflammation during dysbiosis. (**A**–**D**) Water- or AV-treated *Myd88^–/–^* and WT littermate mice were evaluated for foot swelling after CHIKV infection (**A**); immune cell accumulation (**B**); percentages of IFN-γ–, IL-17–, and IL-22–producing CD4^+^ T cells in the joint (**C**); and CHIKV RNA in ipsilateral foot homogenates at 4 dpi (**D**) (2 experiments, *n* = 6–12 per group). (**E** and **F**) Water- or AV-treated *Tlr4^–/–^* and WT littermate mice were assessed for foot swelling after infection (**E**) and immune cell accumulation at 4 dpi (**F**) (2 experiments, *n* = 9–12 per group). (**G**–**I**) Water- or AV-treated WT mice receiving isotype or neutralizing anti–IL-18 mAb were evaluated for foot swelling after infection (**G**), immune cells (**H**), and percentages of IFN-γ–, IL-17–, and IL-22–producing CD4^+^ T cells in the joint at 7 dpi (**I**) (2 experiments, *n* = 7–8 per group). (**J**–**L**) Water- or AV-treated *Villin*-Cre *Myd88^fl/fl^* and littermate Cre^–^ *Myd88^fl/fl^* mice were assessed for foot swelling after infection (**J**), immune cells (**K**), and percentages of IFN-γ–, IL-17–, and IL-22–producing CD4^+^ T cells in the joint at 4 dpi (**L**) (2–3 experiments, *n* = 7–9 per group). (**M**–**O**) Water- or AV-treated *Ccr2*-CreER^T2^ *Myd88^fl/fl^* mice and littermate Cre^–^ *Myd88^fl/fl^* mice were pretreated with tamoxifen and evaluated for foot swelling after infection (**M**), immune cells (**N**), and percentages of IFN-γ–, IL-17–, and IL-22–producing CD4^+^ T cells in the joint at 4 dpi (**O**) (2 experiments, *n* = 5–7 per group). Statistical analysis: **A**, **E**, **G**, **J**, and **M**, 2-way ANOVA with Tukey’s post test or 1-way ANOVA with Dunnett’s post test for AUC; mean values ± SEM. **B**–**D**, **F**, **H**, **I**, **K**, **L**, **N**, and **O**, 1-way ANOVA with Šidák’s post test. ****P < 0.0001, ***P < 0.001, **P < 0.01, *P < 0.05.

We hypothesized that antibiotic-mediated perturbation of the intestinal microbiota and associated permeability promotes joint inflammation via sensing of microbe-derived signals by host pattern recognition receptors. MyD88 is a key adaptor protein linking the TLR and IL-1 receptor families to downstream inflammatory pathways. To assess whether MyD88 signaling contributes to exacerbated inflammation, we treated *Myd88*^–/–^ and WT littermate mice with water or AV prior to CHIKV infection. Loss of MyD88 signaling reversed enhanced foot swelling in AV-treated mice and reduced immune cell infiltration at 4 dpi (Figure 7, A and B). Additionally, IFN-γ, IL-17, and IL-22 production by infiltrating CD4^+^ T cells also was reduced in AV-treated *Myd88*^–/–^ mice (Figure 7C). In contrast, viral titers were slightly but not significantly increased in the feet of *Myd88*^–/–^ mice compared with WT littermate controls (Figure 7D).

We next investigated potential receptors upstream of MyD88 that might trigger increased inflammation in AV-treated CHIKV-infected animals, including TLRs that sense nucleic acid or bacterial cell wall pathogen-associated molecular patterns (PAMPs). We found that enhanced foot swelling and monocyte infiltration were partially reduced in AV-treated *Tlr4*^–/–^ mice (Figure 7, E and F) but not in *Tlr2*^–/–^, *Tlr7*^–/–^, or *Tlr9*^–/–^mice (Supplemental Figure 4, A–C). We also considered the IL-1 and IL-18 signaling pathways. IL-1α/β are cytokines that signal via the IL-1 receptor, which is expressed on a range of cells, including epithelial and immune cells. IL-1 signaling did not contribute to the inflammatory phenotype, as AV-treated *Il1r*^–/–^ and WT mice showed similarly exacerbated foot swelling after CHIKV infection (Supplemental Figure 4D). In contrast, IL-18 was increased in joint-associated, but not intestinal, tissues of AV-treated mice at 7 dpi (Supplemental Figure 4, E and F), and neutralization of IL-18 reduced joint swelling and immune cell infiltration during the second peak of inflammation (Figure 7, G–I). Together, these data suggest that perturbations in the intestinal microbiota result in TLR4-, IL-18-, and MyD88-dependent signals that promote CHIKV arthritis.

MyD88 signaling in intestinal epithelial cells is critical for gut homeostasis and protecting against intestinal bacterial infections (37), but it also can promote extraintestinal inflammation (38, 39). We hypothesized that dysbiosis-induced activation of epithelial cell MyD88 contributes to enhanced joint swelling in AV-treated mice after CHIKV infection. To test this idea, we generated *Villin*-Cre *Myd88*^fl/fl^ mice to delete MyD88 in intestinal epithelial cells. Compared with Cre^–^ littermate controls, intestinal epithelial MyD88 deficiency partially decreased the AV-induced swelling phenotype after CHIKV infection (Figure 7J). Although monocyte infiltration in the ipsilateral foot was unchanged at 4 dpi, there was a small reduction in total CD45^+^ immune cells and neutrophils in AV-treated *Villin*-Cre *Myd88*^fl/fl^ mice (Figure 7K), along with decreased production of IFN-γ and IL-22 by CD4^+^ T cells (Figure 7L). These data suggest that in the context of dysbiosis, MyD88-dependent signaling in intestinal epithelial cells contributes to distal joint inflammation by modulating immune cell recruitment and CD4^+^ T cell responses. Although intestinal tuft cells can sense signals from the microbiota and link them to immune responses (40), improved foot swelling after CHIKV infection was not observed in *Pou2f3*^–/–^ mice, which lack tuft cells (Supplemental Figure 4G).

Given the substantial monocyte infiltration in the joint, we used *Ccr2*-Cre-ER^T2^ *Myd88*^fl/fl^ mice to evaluate whether monocyte-intrinsic MyD88 signaling also contributes to joint inflammation. Following AV treatment, at 3–4 dpi, these mice showed minimal differences in foot swelling, modest but nonsignificant differences in joint-infiltrating immune cells, but decreased IL-17 production by CD4^+^ T cells compared with Cre^–^ littermate controls (Figure 7, M–O). However, during the second phase of disease (7 dpi), AV-treated *Ccr2*-Cre-ER^T2^ *Myd88*^fl/fl^ mice exhibited reduced foot swelling (Figure 7M). Thus, although monocyte accumulation in the joint correlates with enhanced inflammation after AV treatment, the effect is only partially mediated by cell-intrinsic MyD88-dependent activation.

### Monocytes and CD4^+^ T cells are required for exacerbated CHIKV-induced joint swelling in antibiotic-treated mice.

**Figure 8:**
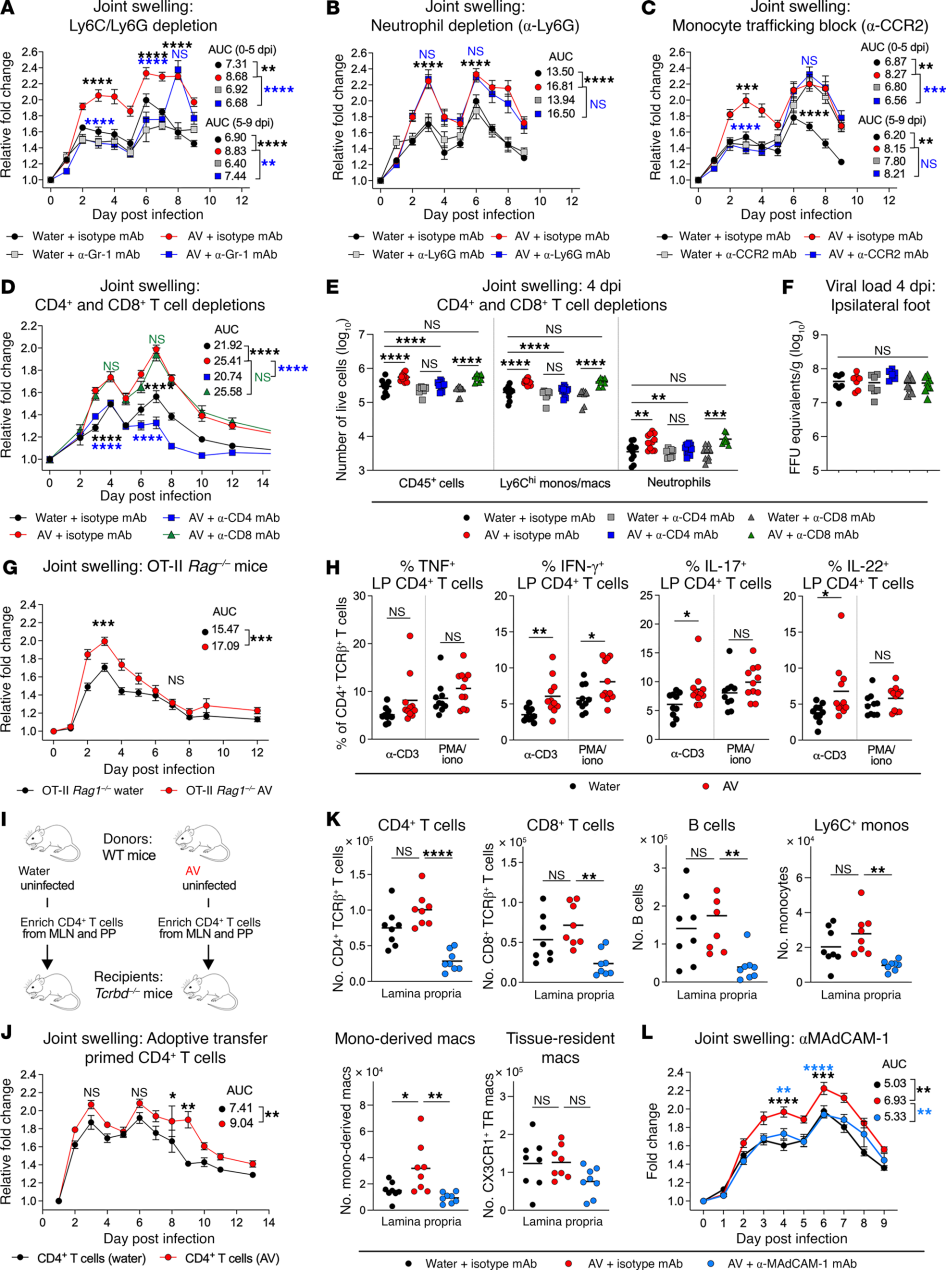
Monocytes and CD4^+^ T cells drive increased joint inflammation associated with gut dysbiosis. (**A**–**C**) Foot swelling after CHIKV infection of water- or AV-treated mice receiving isotype mAb or mAbs against Gr-1 (Ly6C/Ly6G) (3 experiments, *n* = 14–15 per group) (**A**), Ly6G (3 experiments, *n* = 13 per group) (**B**), or CCR2 (3 experiments, *n* = 13–14 per group) (**C**). (**D**–**F**) Mice receiving isotype, anti-CD4, or anti-CD8 mAbs were treated with water or AV and assessed for foot swelling after infection (**D**), immune cells in the joint (**E**), and viral burden in the ipsilateral foot at 4 dpi (**F**) (2–3 experiments, *n* = 7–12 per group). (**G**) Foot swelling after CHIKV infection of water- or AV-treated OT-II *Rag1^–/–^* mice (2 experiments, *n* = 6–7 per group). (**H**) Percentage of CD4^+^ T cells in the colonic lamina propria producing the indicated cytokines. (**I** and **J**) CD4^+^ T cells enriched from MLNs and Peyer’s patches of water- or AV-treated mice were transferred into *Tcrbd^–/–^* mice 1 day prior to CHIKV infection (**I**) and evaluated for foot swelling (**J**). (**K** and **L**) Mice receiving isotype or anti–MAdCAM-1 mAbs prior to treatment with water or AV were evaluated for immune cells in the colonic lamina propria (**K**) and foot swelling after CHIKV infection (**L**) (2 experiments, *n* = 8 per group). Statistical analysis: **A**–**D**, **G**, **J**, and **L**, 2-way ANOVA with Dunnett’s (**A**–**D** and **L**) or Šidák’s (**G** and **J**) post test; AUC analyses were performed with 1-way ANOVA with Dunnett’s post test (**A**–**D** and **L**) or unpaired *t* test (**G** and **J**); mean values ± SEM. **E**, **F**, and **K**, 1-way ANOVA with Šidák’s post test. **H**, unpaired *t* test. ****P < 0.0001; ***P < 0.001; **P < 0.01; *P < 0.05.

To define the cellular drivers of enhanced disease after AV treatment, we depleted specific immune populations prior to CHIKV infection. Broad depletion of myeloid cells (neutrophils, eosinophils, and monocytes) using anti-Ly6C/Ly6G (Gr-1) mAb (Supplemental Figure 4, H and I) reduced foot swelling in AV-treated mice to levels observed in isotype control mAb, water-treated animals (Figure 8A). Whereas depletion of neutrophils alone with anti-Ly6G mAb did not affect CHIKV-induced foot swelling after AV treatment (Figure 8B and Supplemental Figure 4, H and J), inhibition of monocyte trafficking using anti-CCR2 mAb normalized the first swelling peak (Figure 8C and Supplemental Figure 4, H and K). The lack of improvement during the second peak (7 dpi) might reflect compensatory recruitment of neutrophils and eosinophils at the later phase of infection when monocytes and macrophages are depleted (41). In aggregate, these data suggest that circulating monocytes contribute to enhanced CHIKV-induced foot swelling in AV-treated mice.

We also examined the contribution of lymphocytes to enhanced joint swelling after microbiota perturbation. In *Rag1*^–/–^ mice lacking mature T and B cells, AV treatment lost its effect on CHIKV-induced foot swelling (Supplemental Figure 5A). This effect was attributable to T cells, as AV-treated *Tcrbd*^–/–^ mice (lacking both αβ and γδ T cells) did not develop increased foot swelling (Supplemental Figure 5B). In contrast, AV-treated μMT mice that have T cells, but lack mature B cells, developed worsened CHIKV-induced foot swelling (Supplemental Figure 5C), like WT mice (Figure 1G).

To determine the relative contribution of CD4^+^ or CD8^+^ T cells, we depleted these populations with mAbs (Supplemental Figure 5D). Depletion of CD8^+^ T cells had no effect on swelling or immune cell infiltration. However, depletion of CD4^+^ T cells suppressed foot swelling and decreased myeloid cell infiltration in AV-treated animals to levels comparable with water-treated, CHIKV-infected controls (Figure 8, D and E). T cell depletion did not affect the viral load in the ipsilateral feet at 4 dpi (Figure 8F).

We next assessed whether CHIKV-specific CD4^+^ T cells were required for exacerbated joint-associated swelling by treating OT-II *Rag1*^–/–^ mice with antibiotics before infection with CHIKV. In these T cell receptor (TCR) transgenic mice, virtually all CD4^+^ T cells express αβ-TCRs against chicken ovalbumin H-2^b^-restricted peptide 323-339 (42, 43), and there are no CD8^+^ T cells (Supplemental Figure 5E). As in WT mice, AV-treated OT-II *Rag1*^–/–^ mice developed enhanced foot swelling at 4 dpi (Figure 8G), and joint-associated CD4^+^ T cells were capable of producing inflammatory cytokines (Supplemental Figure 5F), suggesting that early antibiotic-driven inflammation is mediated at least in part by antigen-independent, bystander CD4^+^ T cells. These data also suggest that early exacerbation of tissue swelling after AV treatment likely is not due to autoreactive CD4^+^ T cells and molecular mimicry between microbial products and musculoskeletal tissue-associated self-antigens. In contrast, substantially reduced swelling was seen at 7 dpi and thereafter, like that observed in AV-treated *Rag1*^–/–^, *Tcrbd*^–/–^, or CD4^+^ T cell depleted mice (Figure 8D and Supplemental Figure 5, A and B), due to a primary role for CHIKV-specific CD4^+^ T cells in mediating the second swelling peak.

### Gut-associated immune cells are required for increased CHIKV-induced joint swelling in antibiotic-treated mice.

The gut microbiota and SCFA can shape local and peripheral Treg and effector T cell responses (33–35, 44). As mentioned above, we did not detect differences in Treg percentages or numbers in the ipsilateral popliteal lymph nodes or spleens of water- and AV-treated mice at 4 dpi (Figure 3, F–I), or in the spleen prior to infection (Supplemental Figure 5G). To evaluate this question further, we examined the mesenteric lymph nodes (MLNs) that drain the intestines. AV-treated mice showed a reduced proportion of Tregs in the MLN prior to infection, but not at 4 dpi (Supplemental Figure 5H). AV treatment also increased proportions of MLN CD4^+^ T cells producing IFN-γ and IL-17 (Supplemental Figure 5I). Similarly, AV treatment led to a modest increase in the proportion of colonic lamina propria CD4^+^ T cells producing IFN-γ, IL-17, and IL-22 (Figure 8H and Supplemental Figure 6, A and B) without altering the proportions of iNOS- and Arg1-expressing macrophages (Supplemental Figure 6C). These findings suggest that AV-mediated dysbiosis, marked by loss of SCFA and increased gut permeability, alters mucosal CD4^+^ T cell responses.

Immune cells from the gut can migrate to distal sites to promote inflammation (45). To test whether AV-primed gut-associated CD4^+^ T cells are sufficient to drive enhanced joint inflammation, we adoptively transferred CD4^+^ T cells isolated from MLNs and Peyer’s patches of water- or AV-treated uninfected mice into recipient *Tcrbd*^–/–^ mice (Figure 8I). Upon subsequent CHIKV infection 1 day later, *Tcrbd*^–/–^ mice that received CD4^+^ T cells from AV-treated donors developed greater joint swelling than those receiving cells from water-treated controls (Figure 8J). This suggests that gut-associated CD4^+^ T cells shaped by antibiotic treatment can promote enhanced joint inflammation during CHIKV infection.

Finally, we investigated whether preventing immune cell migration to gut-associated tissues could reduce antibiotic-induced exacerbation of joint swelling. Mucosal addressin cell adhesion molecule-1 (MAdCAM-1), expressed on endothelial cells in the intestinal lamina propria, Peyer’s patches, and MLNs, is upregulated by proinflammatory cytokines and mediates α4β7-dependent homing of immune cells to gut-associated tissues (46). Blocking MAdCAM-1 prior to antibiotic treatment reduced immune cell accumulation in the colonic lamina propria (Figure 8K) and MLNs (Supplemental Figure 6D) and attenuated CHIKV-induced joint swelling to levels comparable with those of isotype mAb- and water-treated controls (Figure 8L). Together, these data indicate that immune cells shaped in the gut-associated compartment contribute directly to enhanced CHIKV joint inflammation in antibiotic-treated mice.

### Oral antibiotics alter the transcriptional signature of immune and osteoclast-like cells in joint-associated tissues.

A gap in our understanding of the gut-joint axis is how dysbiosis affects the interplay between tissue-resident and infiltrating immune cells that drive synovial inflammation. To gain insight into this question, we performed scRNA-seq of sorted CD4^+^ T cells, monocytes, and macrophages from ipsilateral joint-associated tissues of mice infected with MAYV that received (a) AV treatment, (b) AV treatment and oral propionate supplementation, (c) AV treatment and CD4^+^ T cell depletion, or (d) water (control) (Supplemental Figure 7, A and B). We used MAYV because it retains the antibiotic-exacerbated joint swelling phenotype (Supplemental Figure 1H), which is rescued by oral propionate supplementation (Supplemental Figure 7C), and it enabled cell sorting at BSL2.

**Figure 9:**
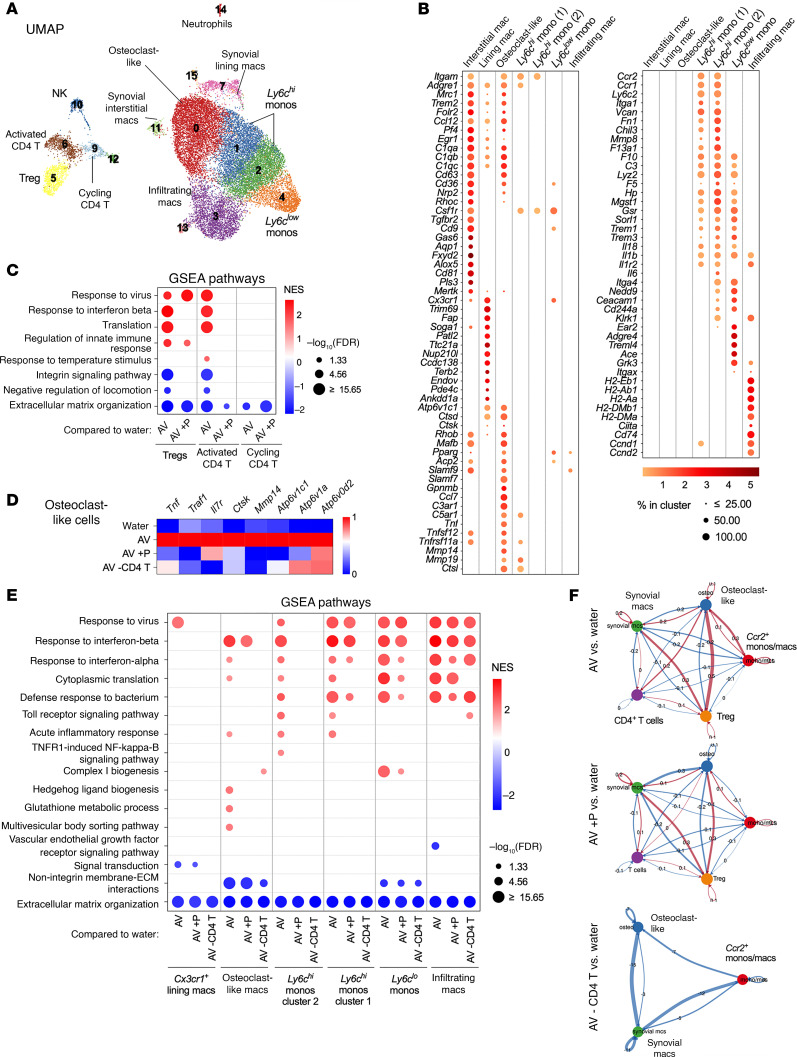
Oral antibiotics alter the transcriptional signature of tissue-resident and infiltrating immune cells in the virus-infected joint. (**A**) UMAP visualization of sort-enriched CD4^+^ T and myeloid cells from scRNA-seq data. (**B**) Bubble plot showing patterns of gene expression among synovial interstitial and lining macrophages, osteoclast-like cells, *Ccr2^+^* monocytes, and monocyte-derived macrophages. (**C**) GSEA pathway analysis of differentially expressed genes in Tregs, activated CD4^+^ T cells, and proliferating CD4^+^ T cells from mice subjected to AV treatment and AV treatment with propionate supplementation (AV + P) compared with corresponding cells from water-treated control mice. NES, normalized enrichment score. (**D**) Heatmap showing expression of osteoclast-related genes in osteoclast-like cells from mice treated with water, AV, AV + P, and AV with CD4^+^ T cell depletion (AV – CD4^+^ T). (**E**) GSEA pathway analysis of differentially expressed genes in tissue-resident and infiltrating monocyte and monocyte-derived macrophages from mice subjected to indicated treatments and compared with water-treated control mice. (**F**) Differential interaction strength between CD4^+^ T cells, tissue-resident synovial macrophages, infiltrating monocytes, monocyte-derived macrophages, and osteoclast-like cells. Line width indicates strength of interaction. Red coloring indicates increased interactions between cell populations in the different AV-treated groups. Blue coloring indicates increased interaction in the water-treated comparator group.

Within joint-associated tissues of MAYV-infected mice, we identified 3 transcriptionally distinct populations of CD4^+^ T cells: Tregs (*Ikzf2^+^ Foxp3^+^ Ctla4^+^*); activated T cells (*Il18r1^+^ Il12rb2^+^ Themis^+^ Nkg7^+^*); and cycling T cells (*Pclaf^+^ Top2a^+^ Stmn1^+^ Mki67^+^* *Cdk1^+^*) (Figure 9A and Supplemental Figure 7, D and E). We also identified 4 populations of infiltrating monocytes and macrophages, including 2 clusters of *Ly6c^hi^ Ccr2^+^ Il1b^+^* monocytes, 1 cluster of *Ly6c^lo^ Ccr2^+^ Il1b^+^* monocytes, and 1 population of *Ccr2^+^ Cd74^+^ Il1b^+^* macrophages (Figure 9, A and B, and Supplemental Figure 7F). Our analysis also revealed populations of tissue-resident synovial lining macrophages (*Cx3cr1^+^ Trim69^+^ Fap^+^*), interstitial macrophages (*Cx3cr1^–^ Trem2^+^ Folr2^+^ Aqp1^+^ Alox5^+^*), and osteoclast-like cells (*Tnfrsf11a^+^ Ctsk^+^ Atp6v0d2^+^ Mmp14^+^ Gpnmb^+^*) (Figure 9, A and B, and Supplemental Figure 7F). These populations have been linked to bone erosion and joint destruction associated with alphavirus-induced arthritis (11–13).

Compared with the corresponding CD4^+^ T cells from water-treated animals, both Tregs and activated *Il18r1^+^ Il12rb2^+^ Nkg7^+^* T cells from joint-associated tissues of AV-treated mice displayed enhanced inflammatory signatures and reduced expression of genes related to integrin signaling and locomotion (Figure 9C). These transcriptional signatures were largely reversed in AV-treated mice that received oral propionate (Figure 9C), consistent with our observation that SCFA treatment reduces proinflammatory cytokine production by joint-associated CD4^+^ T cells.

Osteoclast-like cells from AV-treated mice exhibited increased expression of osteoclast-related genes, including *Ctsk*, *Atp6v1c1*, *Atp6v1a*, *Mmp14*, *Tnf*, and *Il7r* (Figure 9D), along with enrichment of pathways linked to inflammation, Hedgehog ligand biogenesis, glutathione metabolic process, and multivesicular body sorting (Figure 9E), all of which are associated with osteoclast differentiation and bone remodeling (47–50). *Ccr2^+^* infiltrating monocytes and monocyte-derived macrophages from AV-treated mice also exhibited enhanced inflammatory signatures, including increased expression of IFN-stimulated genes (ISGs), TNF family pathways, and bacterial response programs (Figure 9E). In contrast, interstitial macrophages showed minimal transcriptional changes, whereas *Cx3cr1^+^* synovial lining macrophages demonstrated an increased ISG response (Figure 9E). Propionate supplementation of MAYV-infected AV-treated mice largely reversed these transcriptional changes, particularly in synovial lining macrophages, osteoclast-like cells, and some infiltrating monocytes (Figure 9, D and E). Together, these data demonstrate that loss of microbiota-derived SCFAs promotes proinflammatory transcriptional programs in both joint-resident and infiltrating CD4^+^ T and myeloid cells, including upregulation of osteoclastogenic programs, which can be rescued with oral SCFA supplementation.

### Increased inflammatory signatures in myeloid cells are driven by CD4^+^ T cells.

CD4^+^ T cells coexpressing IL-12R and IL-18R are enriched in inflamed synovial tissue from patients with RA (51), and synergistic activation of both receptors induces a IFN-γ response (52). T and myeloid cell crosstalk in the joint can amplify inflammation and promote osteoclast differentiation (53). Given the requirement for CD4^+^ T cells in driving AV-mediated exacerbation of CHIKV arthritis and the presence of activated *Il18r1^+^ Il12rb2^+^ Nkg7^+^* CD4^+^ T cells in joint-associated tissues of MAYV-infected mice, we assessed the contribution of CD4^+^ T cells to the inflammatory signatures of joint-associated macrophages. Indeed, synovial macrophages, infiltrating monocytes, and osteoclast-like cells did not show the increased ISG or proinflammatory signature in AV-treated, MAYV-infected mice lacking CD4^+^ T cells (Figure 9, D and E). Among *Ccr2^+^* infiltrating monocytes, depletion of CD4^+^ T cells had an even stronger effect than propionate supplementation in reversing the AV-driven transcriptional signature, suggesting that the antibiotic-mediated effects on tissue-resident and infiltrating myeloid cells in the joint are mediated principally by CD4^+^ T cells.

**Figure 10:**
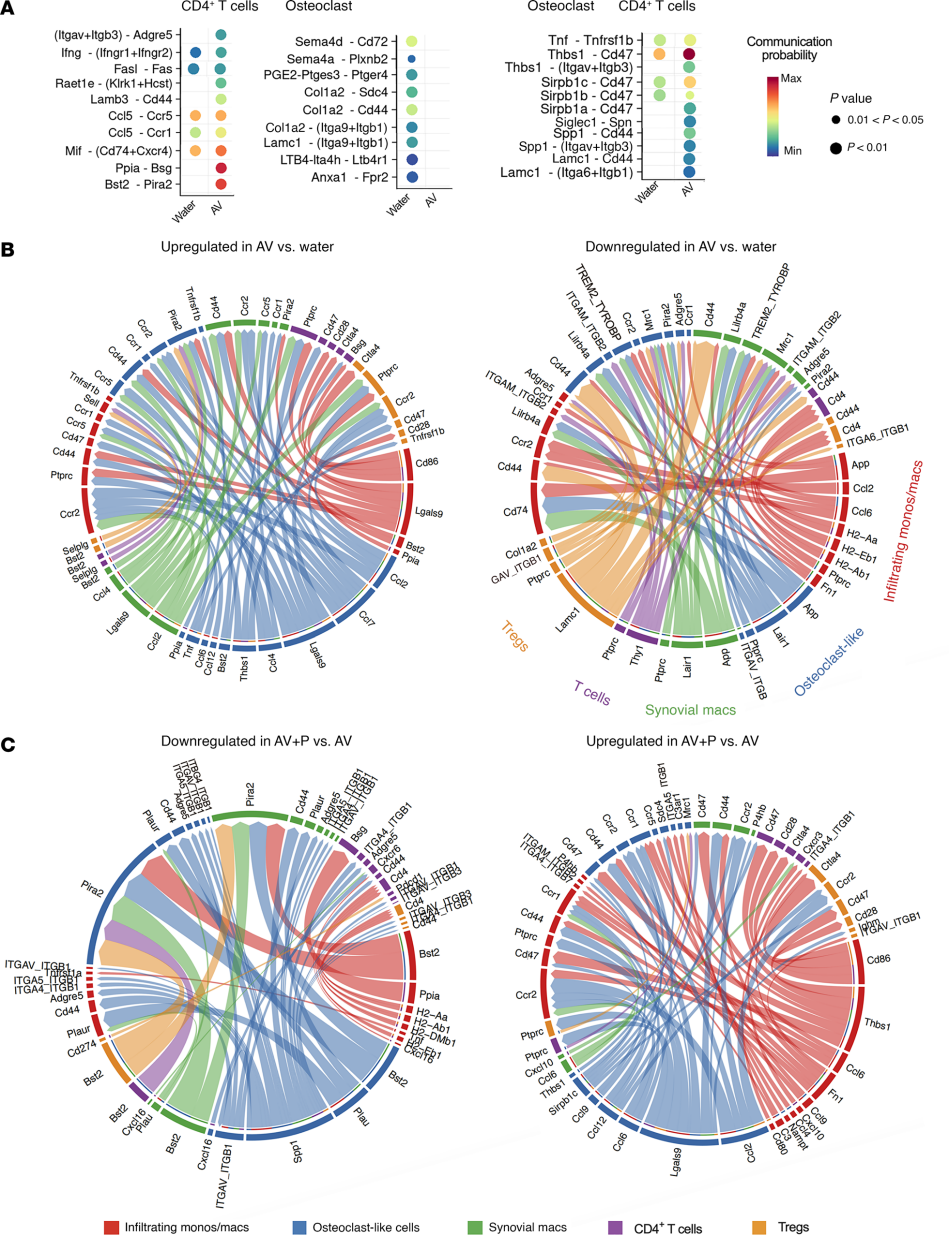
Predicted cell–cell interactions between joint-associated CD4^+^ T and myeloid cells. (**A**) Predicted T cell–osteoclast-like cell interactions based on modeling using CellChat. (**B** and **C**) Chord diagrams of ligand–receptor interactions among Tregs, CD4^+^ T cells, synovial macrophages, osteoclast-like cells, and infiltrating monocytes/macrophages. Shown are the top 60 interactions by weight of up- and downregulated interactions in the joint tissues of MAYV-infected mice that received AV compared with water (**B**) and AV plus propionate (AV + P) compared with AV only (**C**).

Finally, we assessed potential networks of CD4^+^ T cell–myeloid cell interactions in joint tissues of MAYV-infected mice. Cell–cell interaction modeling revealed CD4^+^ T cell communication with myeloid cells in the joint via CCL5-, IFN-γ–, FasL-, and adhesion molecule receptor-mediated interactions (Figure 10A). Activated and cycling CD4^+^ T cell interactions also promote an osteoclastogenic phenotype in infiltrating monocytes/macrophages and osteoclast-like cells through BST2, TSP-1 (*THBS1*), and MIF interactions with their respective receptor ligands (Figure 10A). CellChat-based interaction modeling predicted increased interaction strength between CD4^+^ T cells and osteoclast-like cells, as well as between infiltrating monocytes and osteoclast-like cells, in AV-treated mice (Figure 9F). These interactions were diminished by propionate supplementation or CD4^+^ T cell depletion (Figure 9F). For example, BST2-PIRA2 interactions, which provide critical costimulatory signals for RANK-induced osteoclast differentiation (54, 55), were upregulated in AV-treated mice (Figure 10, A and B) but reduced in AV-treated mice that received propionate (Figure 10C). Our transcriptional profiling data suggest the following: (a) AV-mediated dysbiosis skews CD4^+^ T cells toward an inflammatory signature that activates infiltrating myeloid cells, resident synovial macrophages, and osteoclast-like cells; and (b) this inflammatory phenotype is related to loss of certain SCFAs, such as propionate, and can be reversed with oral propionate supplementation. Thus, we link specific gut microbe-derived SCFAs to intestinal barrier integrity, effects on immune priming, modulation of resident synovial macrophages and osteoclasts, and joint inflammation.

## Discussion

In this study, we establish that antibiotic-induced perturbation of the intestinal microbiota durably exacerbates musculoskeletal inflammation during alphavirus infection via mechanisms distinct from known antiviral effects of gut bacteria (e.g., induction of ISGs, priming of type I and III IFNs, or effects on CD8^+^ T cells) (29, 56–58). Using bacterial and metabolite reconstitution approaches, we link increased joint inflammation to reduced levels of bacterial-derived SCFAs, altered intestinal permeability, and proinflammatory CD4^+^ T cells. When the microbiota is disrupted, MyD88 signaling in intestinal epithelial cells and monocytes drives accumulation of inflammatory monocytes and polarized CD4^+^ T cells in the joint, promoting differentiation of osteoclast-like cells. Thus, intestinal microbes and their metabolites can regulate intestinal epithelium and immune cells and shape the severity of alphavirus-induced inflammation in the joint.

Despite evidence supporting a gut-joint axis (8, 9, 59), the mechanisms linking intestinal and joint inflammation remain unclear. In humans, intestinal inflammation and infection can trigger joint inflammation, including reactive and inflammatory bowel disease–associated arthritis (60). Although these conditions have been linked to HLA-B27, the IL-23/IL-17 axis, and adhesion receptors, the underlying mechanisms are not defined (61). Epidemiological studies also link antibiotic usage to increased risk of RA and juvenile idiopathic arthritis (1–6). Gut dysbiosis, including loss of butyrate-producing bacteria, has been observed in RA (62–64) and is thought to promote disease through disruption of intestinal barrier function, dysregulation of proinflammatory cytokines, altered Tfh cell responses, and autoantibody production. Notably, treatment with disease-modifying antirheumatic drugs or TNF inhibitors can modulate dysbiosis (65–67), suggesting bidirectional interactions between systemic inflammation and the gut microbiota. Similarly, CHIKV infection of rhesus macaques alters gut microbial composition and metabolites, with increased proinflammatory gene expression in GI tract tissues, including the inflammasome and IL-17 pathways (68).

Our experiments demonstrated that antibiotic-induced gut dysbiosis increased musculoskeletal inflammation after CHIKV infection through inflammatory monocytes and both bystander and antigen-specific effector CD4^+^ T cells. While these cell types are known contributors to CHIKV-induced joint inflammation (14, 17–19, 69–72), our study revealed a distinct requirement for proinflammatory CD4^+^ T cells in exacerbating arthritis in the context of gut dysbiosis. This finding highlights an important role of the gut microbiota and its metabolites in shaping effector T cell phenotypes during arthritis, influencing both infiltrating and synovial tissue-resident macrophages. Unlike studies using prolonged broad-spectrum antibiotics, a 3-day oral AV regimen did not reduce peripheral Tregs, either at baseline or after CHIVK infection. Rather, short-term dysbiosis increased proinflammatory CD4^+^ T cells and infiltrating monocytes in joint-associated tissues after CHIKV infection. Neutralization of TNF, IL-17A, IFN-γ, or IL-18 improved joint swelling in AV-treated mice to varying degrees.

Oral antibiotics exacerbated CHIKV arthritis in a MyD88-dependent manner, with a partial contribution from gut epithelium-specific MyD88 signaling. The effect was partially dependent on TLR4 signaling, while no significant change in joint-associated swelling was observed in antibiotic-treated mice with individual deficiencies in IL-1R, TLR2, TLR7, or TLR9. Notably, *Myd88*^–/–^ mice showed a greater reduction in swelling than any single TLR-deficient strain, suggesting that multiple PAMPs may drive inflammation, which could potentially mask the effects of individual TLRs. Alternatively, other TLRs or MyD88-dependent non-TLR signaling pathways may also play a role.

Intestinal epithelial MyD88 signaling is essential in gut homeostasis through its function in maintaining the epithelial barrier, intestinal mucus production, and mucosa-associated antimicrobial activity (37). However, our results show that, under conditions of microbiota perturbation, enterocyte MyD88 signaling promotes distal musculoskeletal inflammation, potentially by enhancing immune activation, as described for ischemia/reperfusion-induced intestinal injury (73). Epithelial MyD88 signaling has also been linked to renal injury (39) as well as diet-induced obesity and hepatic steatosis (38), although the contribution of microbial factors in these settings remains unclear.

Disruption of the gut barrier can drive inflammation both locally and at distal sites through the migration of activated immune cells or translocation of PAMPs into circulation. These signals may activate peripheral immune cells through MyD88-dependent and -independent pathways to amplify inflammation. However, the minimal protection conferred by monocyte-specific MyD88 deletion, together with the role of CD4^+^ T cells in promoting monocyte infiltration and activation in the joint, suggests that monocyte-intrinsic MyD88 signaling contributes only partially to CHIKV-induced musculoskeletal inflammation under dysbiotic conditions. Further investigation is needed to define how epithelial MyD88 signaling transduces luminal cues to shape effector T cell responses and regulate local and systemic inflammation.

We identified specific anaerobic bacteria (e.g., *C*. *scindens* and *B*. *thetaiotaomicron*) that mitigate the deleterious effects of microbiota perturbation on CHIKV-induced musculoskeletal inflammation. The protective effect of *B*. *thetaiotaomicron* is mediated through production of its primary SCFA, propionate, suggesting that multiple bacterial species across different phyla may contribute to antiinflammatory effects during systemic viral infection through immunoregulatory metabolites. Although exogenous butyrate has been shown to support intestinal barrier integrity and reduce disease severity in sterile arthritis models (74, 75), the upstream and downstream mediators have remained unclear. Our findings establish a role for an endogenously produced SCFA in modulating virus-induced arthritis and define the epithelial and immune cell interactions — as well as cell-intrinsic and -extrinsic signaling pathways — that link the gut microbiota to joint inflammation.

In viral arthritis, SCFAs may act locally at the intestinal interface or enter circulation to influence responses at distal sites of inflammation. Locally, SCFAs support gut barrier integrity and induce intestinal Tregs (33, 34), while circulating SCFAs can support peripheral Treg differentiation (35). In contrast to reports showing that oral butyrate exacerbates CHIKV arthritis in mice (36), we did not observe effects of exogenous SCFAs on CHIKV-induced joint inflammation in mice with an intact microbiota. The difference in CHIKV arthritis outcomes may reflect dose-dependent effects of oral butyrate (200 mM in ref. 36 versus 37 mM in our study) on epithelial function and immune regulation: lower doses promote colonocyte proliferation and barrier function, whereas higher doses can increase intestinal epithelial permeability and inflammation (76).

Our experiments showed that (a) CD4^+^ T cells from MLNs and Peyer’s patches of antibiotic-treated mice can transfer an enhanced CHIKV-induced joint swelling phenotype to untreated *Tcrbd^–/–^* recipients, and (b) blocking immune cell homing to gut-associated tissues prior to antibiotic treatment reduces the impact of antibiotics on joint inflammation. These data support the conclusion that activated gut-associated immune cells — including CD4^+^ T cells — are necessary and sufficient to drive joint inflammation in the context of gut dysbiosis.

Macrophages are regulators of tissue homeostasis, inflammation, and cartilage/bone damage, and synovial macrophage changes often precede disease in inflammatory arthritis (77, 78). Bidirectional crosstalk between T cells and monocytes/macrophages further amplifies inflammation and arthritis severity (53). To our knowledge, how the gut microbiota shapes synovial macrophage interactions in the joint had not been explored. We found that antibiotic-induced loss of SCFAs led to increased infiltration of proinflammatory monocytes/macrophages into alphavirus-infected joints, an enhanced ISG signature in CX3CR1^+^ synovial lining macrophages, and expansion and transcriptional activation of osteoclast-like cells, the latter of which are linked to bone erosion, a feature of alphavirus disease (11–13, 69, 79). These pathological changes were attenuated by propionate supplementation or CD4^+^ T cell depletion, highlighting the coordinated role of microbial metabolites, gut-primed T cells, and T cell–macrophage interactions in regulating joint inflammation and tissue damage.

In summary, our study defines a mechanistic link between antibiotic-induced disruption of commensal gut microbes, loss of microbial-derived SCFAs that maintain epithelial and immune cell homeostasis, and dysregulated immune responses that drive acute alphavirus arthritis. SCFA-producing bacteria across multiple phyla maintain equilibrium between immune tolerance and host defense, which can be disrupted even by short-term antibiotic exposure. Our work reveals an underrecognized role for microbiota-derived SCFAs in restraining effector and bystander CD4^+^ T cell polarization, thereby limiting inflammatory monocyte accumulation, the proinflammatory reprogramming of synovial tissue-resident macrophages, and osteoclast-like cell differentiation in the joints. These results highlight the gut microbiota, their metabolites, and downstream host signaling pathways as potential therapeutic targets for viral and noninfectious inflammatory arthritis.

## Methods

Additional details may be found in Supplemental Methods.

### Sex as a biological variable.

Both male and female mice were studied, and similar findings are reported for both sexes.

### Conventionally housed mice.

Specific pathogen–free C57BL6/J mice (strain 000664) were obtained from The Jackson Laboratory. *Rag1^–/–^* (*Rag1^tm1Mom^*, 034159), *Tcrbd^–/–^* (*Tcrb^tm1Mom^ Tcrd^tm1Mom^*, 002122), *μMT* (*Ighm^tm1Cgn^*, 002288), *Myd88^–/–^* (*MyD88^tm1.1Defr^*, 009088), *Il1r^–/–^* (*Il1r1^tm1Imx^*, 003245), *Tlr7^–/–^* (*Tlr7^tm1Flv^*, 008380), *Tlr9^–/–^* (*Tlr9^M7Btlr^*, 34329-Jax), *Tlr2^–/–^* (*Tlr2^tm1Kir^*, 004650), *Tlr4^–/–^* (*Tlr4^tm1.2Karp^*, 029015), and *Pou2f3^–/–^* (*Pou2f3^em1Cbwi^*, 037040) mice were purchased from The Jackson Laboratory and bred as heterozygotes to generate KO and WT littermate control mice. CD45.1 OT-II *Rag1^–/–^* mice were maintained using homozygote crosses. Mice lacking MyD88 expression in intestinal epithelial cells and monocytes were generated by crossing *Myd88^fl/fl^* mice (strain 008888) with *Villin*-Cre (strain 004586) or *Ccr2-*Cre-ER^T2^ mice (strain 035229), all obtained from The Jackson Laboratory. Mice were housed in enhanced caging that was preassembled prior to autoclaving, provided an autoclaved 20% protein diet and water ad libitum, and maintained on a 12 h light/12 h dark cycle.

### Gnotobiotic mice.

C57BL/6J GF mice were bred and maintained at the Washington University Gnotobiotic Core Facility. GF status was confirmed by 16s qPCR analysis (Charles River).

### Viruses.

Stocks of CHIKV La Reunion OPY1 were generated from an infectious cDNA clone using published methods (80). MAYV BeH407 was obtained from the World Reference Center for Emerging Viruses and Arboviruses. Viruses were grown in Vero CCL-81 cells and titrated by focus-forming assays.

### Virus infection and assessment of foot swelling.

3- to 4-week-old mice were anesthetized with ketamine hydrochloride (80 mg/kg)/xylazine (15 mg/kg) prior to subcutaneous infection with 10^3^ focus-forming units of CHIKV-LR or MAYV in a 10 μL volume in the left rear footpad. The mediolateral aspect of the tarsus and dorsal-ventral aspect of the ipsilateral foot were measured using digital calipers.

### Antibiotic treatment, SCFA or DCA supplementation, and tamoxifen administration.

3- to 4-week-old mice were provided with water or antibiotics (0.5 g/L vancomycin and/or 1 g/L ampicillin) in drinking water ad libitum for 3 days. For oral metabolite supplementation, SCFAs (37 mM sodium butyrate, sodium propionate, or sodium acetate; Sigma-Aldrich) or DCA (0.2% [w/v] sodium deoxycholate; Sigma-Aldrich) were added to drinking water at the time of antibiotics initiation and continued after their cessation. For Cre induction, tamoxifen (Sigma-Aldrich) was dissolved in safflower oil and administered to 3- to 4-week-old *Myd88*^fl/fl^ *Ccr2* Cre-ER^T2^ or *Myd88*^fl/fl^ littermate control mice via intraperitoneal injection at a dose of 75 mg/kg daily for 3 days prior to initiation of antibiotics.

### Fecal microbial transfer, bacterial culture, and bacterial reconstitution.

For FMT stocks, cecal contents were diluted in PBS with 10% glycerol, homogenized, and passed through a 100 μm nylon filter. For bulk FMT-derived aerobic and anaerobic bacteria, FMT stocks were cultured in liquid Brain Heart Infusion (BHI) medium under aerobic or anaerobic conditions, respectively. *C*. *scindens* and *B*. *thetaiotaomicron* were cultured in BHI or chopped meat media broth under anaerobic conditions. For bacterial reconstitution experiments, mice were rested for 1 day after discontinuation of antibiotics prior to oral gavage with sterile PBS or with 10^8^ colony-forming units of bacteria in a 100 μL volume.

### Cytokine neutralization.

Mice were treated with neutralizing mAbs against TNF (200 μg; clone MP6-XT22, BioLegend), IFN-γ (100 μg; clone H22, Leinco Technologies), IL-17A (100 μg; clone 17F3, Bio X Cell), IL-18 (150 μg; clone YIGIF74-1G7, Bio X Cell), or isotype control mAb every 3 days starting 1 week prior to infection and continued after infection.

### Statistical analysis.

Statistical significance was assigned when *P* values were < 0.05 using GraphPad Prism version 9.3. Tests, number of animals, mean values, and statistical comparison groups are indicated in the figure legends.

### Study approval.

Housing and care of laboratory animals were conducted in accordance with guidelines from the NIH Guide for the Care and Use of Laboratory Animals. Protocols were approved by the Institutional Animal Care and Use Committee at the Washington University School of Medicine (assurance number A3381-01).

### Data availability.

All data supporting the findings of this study are available within the paper or from the corresponding author. Data supporting the graphs are provided in the Supporting Data Values file. 16s rRNA and scRNA-seq data are available at the European Nucleotide Archive (project accession: PRJEB53328) and the Gene Expression Omnibus (project accession: GSE288863), respectively.

## Author contributions

FRZ, ESW, HJ, RBW, LD, TTL, LH, and CJG performed experimental studies. FRZ, MK, ESW, RBW, CJG, LW, AJ, SAH, MAF, MNA, and MSD analyzed the data. CSH, MM, MTB, TSS, and LBT provided critical reagents and input on experimental design. FRZ and MSD designed research studies and wrote the initial draft of the manuscript.

## Conflict of interest

MSD reports personal consulting or advisory fees from Inbios, Akagera Medicines, Merck, IntegerBio, and GlaxoSmithKline and grants from Moderna.

## Funding support

This work is the result of NIH funding, in whole or in part, and is subject to the NIH Public Access Policy. Through acceptance of this federal funding, the NIH has been given a right to make the work publicly available in PubMed Central.

- NIH grants R01 AI152484 to MSD, R01 DK132244 and R01 DK135816 to CJG, T32 CA009547 and K08 AR084597 to FRZ, F30 AI152327 to ESW, and P30 AR074992 to Washington University Musculoskeletal Research Center.
- Systems Immunology Initiative award by the Department of Pathology & Immunology of Washington University School of Medicine (to MNA).
- American Gastroenterological Association (to CJG).
- W.M. Keck Foundation (to CJG).
- RAPP funding from Weill Cornell Medicine (to CJG).
- Rheumatology Research Foundation (to FRZ).

## Supplementary Material

Supplemental data

Supporting data values

## Version 1 07/15/2026

Electronic publication
