# Supplementary material for: Gut microbe–derived short-chain fatty acids regulate alphavirus arthritis and macrophage activation in mice
Source: J Clin Invest. 2026 Jul 15;136(14):e202262. doi: 10.1172/JCI202262 (PMC13367971; doi:10.1172/JCI202262)
Supplement: Supplemental data [file jci-136-202262-s145.pdf]

SUPPLEMENTARY METHODS, REFERENCES, FIGURES, TABLES, AND LEGENDS

SUPPLEMENTARY METHODS

T cell, monocyte, and neutrophil depletion or blockade of immune trafficking. For T cell depletion, mice were treated with 25 μg anti-CD4 (clone GK1.5; BioXCell), anti-CD8 (clone 2.43; BioXCell), or isotype mAb (clone LTF-2; BioXCell) via intraperitoneal injection every 3 days for 1 week prior to and up to 2 weeks after infection. To target myeloid cells, mice were treated via intraperitoneal injection one day prior to infection and every 2 days afterwards with the following: combined monocyte, eosinophil, and neutrophil depletion [250 μg anti-Ly6G/Ly6C (Gr-1; clone RB6-8C5) or isotype mAb (clone LTF-2; BioXCell)]; neutrophil-only depletion [250 μg anti-Ly6G (clone 1A8; BioXCell) or isotype mAb (clone 2A3; BioXCell)]; or blockade of monocyte trafficking [25 μg anti-CCR2 (clone MC-21) (1) or isotype control mAb (clone LTF-2; BioXCell)]. Depletions were confirmed by flow cytometric analysis of immune cell populations in the blood. For blockade of immune cell migration to gut-associated tissues, mice were treated with 100 μg anti-MAdCAM-1 (clone MECA-367; BioXCell) or isotype control mAb (clone 2A3; BioXCell) via intraperitoneal injection one day prior to initiation of antibiotics and then every 3 days afterwards. Blockade was confirmed by flow cytometric analysis of immune cell populations in the colonic lamina propria and MLN.

Histology and viral RNA in situ hybridization. Ipsilateral feet were collected after extensive perfusion with PBS and 4% paraformaldehyde (PFA), fixed in 4% PFA for 24 h, decalcified in 14% EDTA free acid (Sigma-Aldrich) at pH 7.2 for 10 days, and dehydrated in 70% ethanol. Paraffin-embedded tissues were sectioned; hematoxylin and eosin, TRAP, and Toluidine blue staining were performed following standard procedures. Viral RNA in situ hybridization was performed using RNAscope 2.5 (Advanced Cell Diagnostics) according to manufacturer's instructions using the CHIKV probe (479501).

Viral burden analysis. Ipsilateral feet were collected from CHIKV-infected mice following extensive perfusion with PBS, weighed and homogenized in 500 µL of PBS with zirconia beads in a MagNA Lyser (Roche), followed by centrifugation of homogenates at 10,000 rpm for 5 min at 4°C. Viral burden was determined by focus forming assay on Vero CCL-81 cells as described (2). For some samples, RNA was extracted using a MagMAX *mir*Vana Total RNA isolation kit (Thermo Scientific) and a Kingfisher extraction machine (Thermo Scientific). MAYV RNA levels for tissue homogenates and viral stocks were determined using previously designed primer/probe sets for MAYV (2) by one-step quantitative reverse transcriptase PCR with Taqman RNA-to-Ct 1-step kit (Thermo Scientific) on an ABI 7500 Fast Instrument using standard cycling conditions. Viral titers are expressed on a log_{10} scale as FFU equivalents per gram of tissue, determined by comparing MAYV RNA equivalents on a standard curve using RNA isolated from known viral stocks.

***In vivo* intestinal permeability assay.** Mice were administered 100 µL of 60 mg/mL dextran of different molecular weights, each conjugated to different fluorescent dyes (CascadeBlue 10 kDa, Thermo; tetramethylrhodamine 70 kDa, Thermo; FITC 250 kDa, Sigma-Aldrich; FITC 2000 kDa, Sigma-Aldrich). Blood was collected into EDTA tubes after 1.5 and 4 h and centrifuged (1500g for 10 min at 4°C). Plasma was diluted 1:1 with phosphate buffered saline (PBS) and plated in triplicates. Fluorescence was measured with a BioTek Synergy H1 plate reader, and concentrations were calculated based on a standard curve.

Adoptive transfer of CD4$^+$ T cells from MLN and Peyer's patches into *Tcrbd*$^{-/-}$ mice. MLN and Peyer's patches were collected from water- or AV-treated mice. Single cell suspensions were generated via mechanical dissociation through a 70 µm filter, and CD4$^+$ T cells were purified by negative selection using the mouse CD4$^+$ T cell isolation kit (Miltenyi Biotec) following the manufacturer's instructions. Isolated T cells were pooled, and 10^6 CD4$^+$ T cells from either water- or AV-treated donors were adoptively transferred via retro-orbital injection into *Tcrbd*$^{-/-}$ recipients. Mice were infected with CHIKV at one day after T cell transfer.

Cytokine and chemokine analysis. Cytokine and chemokine analysis of homogenized joint tissues was performed by Eve Technologies using a Millipore Mouse Cytokine Array/Chemokine Array platform (virus was inactivated using 1% Triton-X-100). Alternatively, cytokines (IFN-γ, TNF, and IL-17A/F) were measured from tissue homogenates prior to virus inactivation by ELISA (Biolegend) following the manufacturer's instructions. IL-18 levels in homogenates of gut or joint tissues were measured using a Luminex 200 system (Immunomonitoring Lab at Washington University School of Medicine) and the IL-18 Mouse ProcartaPlex™ Simplex Kit (ThermoFisher) per the manufacturer's instructions.

Generation and characterization of *B. thetaiotaomicron* mutants. *Vector assembly.* To generate the propionate deficient mutant, the conventional double-crossover gene deletion method was utilized (3) to knock out *mmdA* genes (BT_2090 and BT_2091) of *B. thetaiotaomicron*. First, two fragments with a length of ~1200 bp flanking the target gene were amplified and fused via fusion PCR. This fragment was assembled with a PCR-amplified *pExchange-tdk* (3) backbone using Gibson Assembly and transformed into *E. coli* S17 directly to form a suicide vector that is introduced into *B. thetaiotaomicron* by conjugation described below. All primers used in the cloning are listed in **Supplemental Table 3**.

Introducing vectors by conjugation into B. thetaiotaomicron. The suicide vectors were introduced into *B. thetaiotaomicron* based on a published method (4). First, a single colony of *B. thetaiotaomicron Δtdk* (a strain with the *BT_2275* gene, which encodes a deleted thymidine kinase gene) was inoculated in 3 mL of TYGB broth culture in an anaerobic chamber at 37°C under an atmosphere consisting of 10% CO_2, 5% H_2, 85% N_2 for 12 h. *E. coli* S17 harboring a suicide vector were grown in 3 mL of LB broth supplemented with carbenicillin (100 µg/mL) at 37°C with shaking at 225 rpm. After ~10 to 12 h, when the OD600 of *E. coli* S17 reached 0.8-1.0, 3 mL of *E. coli* S17 culture was centrifuged at 1500 x g for 3 min. The supernatant was discarded, and the cell pellet was washed once with 1.5 mL of PBS (pH 7.4). The washed *E. coli* S17 cell pellet was resuspended in a 3-mL overnight culture of *B. thetaiotaomicron*, gently mixed by pipetting, and

passed through a 0.2 µm filter. The liquid was discarded, and the filter membrane with the mixture of donor and recipient cells was placed onto the surface of a pre-reduced TSAB plate. The plate was incubated aerobically in a 37°C incubator for 24 h. The filter membrane was soaked in 2 mL of pre-reduced TYGB medium. The cells on the filter were resuspended into the medium by vortexing. The mixture was then transferred into the anaerobic chamber, and 100 µL was plated onto a pre-reduced TSAB plate + 200 µg/mL gentamycin + 25 µg/mL erythromycin. Colonies with plasmid inserted into the genome typically appeared after 36-48 h. Eight of these colonies were picked and re-streaked on pre-reduced TSAB plate + 200 µg/mL gentamycin + 15 µg/mL thiamphenicol to isolate single colonies. After 1 to 2 days, a single colony of each single crossover recombinant was cultured into TYGB medium, grown overnight for 16-24 h, and combined and plated on TSAB plates containing FUdR (200 µg/ml). Ten FUdR-resistant colonies were re-streaked to single colonies and subject to diagnostic PCR using the primers listed in **Supplemental Table 3**.

LC-MS analysis of propionate in liquid culture. Single colonies of wide-type or mutant bacterial strains were inoculated in BHI+ Medium and grown anaerobically at 37°C. After 24 h, supernatant of each sample was collected for LC-MS analysis. 20 µL of supernatant of liquid culture was mixed with 200 µL of SCFA derivatization solution (freshly prepared acetonitrile solution containing 1 mM 2,2′-dipyridyl disulfide (DPDS), 1 mM triphenylphosphine (TPP) and 1 mM 2-hydrazinoquinoline (HQ)). The resulting mixture was vortexed and incubated at 60°C for 1 h, centrifuged at 21,000 g for 20 min, and its supernatant was analyzed by LC-MS. We used the following solvent system for detection of SCFA derivatives: A: H_2O with 0.1% formic acid; B: methanol with 0.1% formic acid. 1 µL of each sample was injected with a flow rate of 0.35 ml/min and a column temperature of 40°C. The gradient for HPLC-MS analysis was: 0 - 6.0 min 99.5%A – 70.0%A, 6.0 - 9.0 min 70.0%A – 2.0%A, 9.0 - 9.4 min 2.0%A – 2.0%A, 9.4 - 9.6 min 2.0%A – 99.5%A. Peaks were assigned by comparison with authentic standards.

In vitro growth curve of B. theta WT and mutant strain. Three single colonies of wide-type or mutant strains were inoculated in BHI+ Medium and anaerobically cultured for overnight at 37°C to reach late-log phase. Then, 2 µL of the culture was resuspended in 200 µL of BHI+ broth, loaded into a 96-well plate, and incubated anaerobically at 37°C in a Multiskan SkyHigh Microplate Spectrophotometer (Thermo Fisher). OD600 nm readings were recorded every 30 min for 30 h.

Quantification of fecal *Bacteroides*. Fecal samples from mice treated with AV and gavaged with sterile PBS, or wild-type or mutant *B. thetaiotaomicron,* were collected in sterile microcentrifuge tubes, stored on ice, and transferred to an anaerobic chamber within 1 h of collection. A volume of 500 µL of sterile PBS was added to each tube, and samples were subjected to mechanical disruption via vortexing and pipetting. Ten-fold serial dilutions were cultured on *Bacteroides* bile esculin agar and counted after 48 h to determine colony-forming units.

Gas chromatography mass spectrometry for SCFA measurements. Cecal samples were collected and frozen on dry ice prior to storage at -80°C. Samples (30 mg) were suspended in 300 µL of extraction solvent (1-pentanol and H_2SO_4, with an internal standard of d7-butyric acid) and homogenized by vortexing and sonication. For standards, pentyl esters of d7-Butyric acid (Cayman Chemical Company), butyric acid (Alfa Aesar), propionic acid (Sigma-Aldrich), and acetic acid (Fluka), were prepared and subjected to the same extraction procedure. All samples were incubated for 2 h at 80°C while mixing at 1,200 rpm, before cooling to room temperature. 300 µL of 0.9% NaCl and 300 µL of hexane were added to each sample. Samples were then vortexed for 20 sec, mixed for 2 min at 1,200 rpm at room temperature, and then centrifuged for 2 min at 13,200 rpm. 100 µL of the organic phase (supernatant) was transferred to a gas chromatography (GC) vial with insert for analysis using 1 µL injections in triplicate. For GC mass spectrometric analysis, an Agilent 7890A GC equipped with a 5975c MS detector (He carrier gas) was used. Inlet settings: 250°C, 17.8 psi, total flow 14.2 mL/min, and split ratio 10:1. Column: Phenomenex Zebron ZB-5MSi Guardian fused silica capillary column (30 m x 0.25 mm x 0.25 µm

film thickness), 1.2 mL/min flowrate, 17.8 psi, constant flow. Oven: initial temperature 85°C hold for 4 min, ramp 1 to 110°C at 1 °C/min hold for 0 min, ramp 2 to 300 °C at 85°C/min hold for 2 min, ramp 3 to 85 °C/min hold for 1.2 min, total run time 36.2 min. Mass spectrometry (MS) conditions: transfer line 300°C, MS source 230°C, MS quad 150°C electron energy 70.3 eV. Data were visualized as EIC with *m/z* 61, 75, 89, and 96 observed for characteristic fragments from pentyl acetate, pentyl propionate, pentyl butyrate, and d7-pentyl butyrate (IS), respectively.

Flow cytometry. Single cell suspensions were obtained from spleens and lymph nodes by mechanical dissociation, followed by filtration through 70 µm nylon mesh filters. For colonic lamina propria, mice underwent terminal anesthesia and extensive perfusion with PBS. The colon was collected and flushed with cold PBS, and surrounding mesentery and fat were removed. The colon then was cut longitudinally and washed again in Hank's balanced salt solution (HBSS) containing 15 mM HEPES. To remove the epithelium, tissue pieces were incubated in HBSS containing 1 mM DTT and 15 mM HEPES for 20 min at 37°C with rotation, followed by incubation in HBSS containing 5 mM EDTA and 15 mM HEPES for 20 min at 37°C with rotation. To isolate lamina propria cells, the remaining tissue pieces were rinsed by sequential swishing in Petri dishes containing HBSS with 15 mM HEPES, then digested in complete RPMI (10% FBS, 2 mM L-glutamine, 1 mM sodium pyruvate, 50 µM beta-mercaptoethanol, and penicillin/streptomycin) with 100 U/mL collagenase IV for 1 h at 37°C with rotation. Digested samples were filtered over 100 µm nylon mesh filters and centrifuged at 850 x g for 10 min. Immune cells in the lamina propria were enriched by 70/40% Percoll gradient centrifugation at 850 x g for 20 min (no brake) with cells collected from the interface. For ipsilateral foot specimens, tissues were grossly dissected after perfusion, followed by digestion in RPMI with 10% FBS, 15 mM HEPES, type I collagenase (Thermo; 2.5 mg/mL) and DNase (Sigma-Aldrich; 10 µg/mL) for 1 h at 37°C with gentle agitation, followed by filtration through a 70 µm nylon filter. After blocking with TruStain FcX™ PLUS (anti-mouse CD16/32, Biolegend), cells were stained with primary antibodies in PBS with 2% FBS. For intracellular staining, cells were fixed and permeabilized using the

FoxP3/Transcription Factor Staining Buffer Set (eBioscience) following manufacturer's instructions and stained overnight at 4°C with secondary antibody in permeabilization buffer. For intracellular cytokine analysis of T cells (including from the joint), cells were incubated ex vivo at 37°C for 6 h in the presence of brefeldin A (BD Biosciences) in RPMI supplemented with 10% FBS, 2 mM L-glutamine, 1 mM sodium pyruvate, and 50 μM beta-mercaptoethanol, and left either unstimulated or stimulated with 1 μg/mL of plate-bound anti-CD3/CD28 or PMA (50 ng/mL)/ ionomycin (1 μM). For cytokine production by joint-associated T cells, data from the anti-CD3/CD28 treatment condition is shown. For CD107a-cycling experiments, conjugated antibodies against CD107a were included in the medium during the incubation period as a measure of T cell degranulation. Subsequently, surface and intracellular staining of cells was performed as detailed above. Fluorescent conjugated antibodies used for flow cytometry staining are listed in **Supplemental Table 4**. Samples were processed on a BD LSRFortessa X-20 flow cytometer (BD Biosciences) and analyzed using FlowJo software.

Cell sort enrichment of $CD4^+$ T cells, monocytes, and macrophages for single cell sequencing. Mice were treated with water, AV, AV supplemented with propionate, or AV after $CD4^+$ T cell depletion (4 mice per group) prior to infection with MAYV. At 5 dpi, tissues from the ipsilateral feet were grossly dissected, and single cell suspensions were obtained by digestion with gentle agitation as described above. Cells were blocked with TruStain FcX™ PLUS (anti-mouse CD16/32, Biolegend) and then stained with TotalSeq anti-mouse hashtag antibodies (Biolegend B0302, B0304, B0306, B0308; one distinct hastag per mouse sample in each group following manufacturer's instructions) and fluorescently conjugated antibodies against cell surface antigens. After washing, individual samples were pooled according to experimental group and resuspended in sorting buffer (PBS with 1% FBS, 1mM EDTA, and 25 mM HEPES). Fluorescence activated cell sorting was performed using a BD FACSAria II flow cytometer (BD Biosciences). Sorted cell populations were pooled according to experimental group and resuspended in PBS with 0.04% BSA prior to processing for single cell RNA sequencing.

Single cell RNA sequencing and analysis. The Cell Ranger multi pipeline version 8.0.1, available on the 10x Genomics website, was utilized for demultiplexing pulled samples and aligning reads to the mouse reference genome mm10-2020-A. On average, 92.9% of reads per sample were successfully mapped, with over 90.5% of reads having a quality score above q30. Cells assigned one single CMO hashtag were analyzed, resulting in 27,839 cells (9,251 + 5,825 + 7,681 + 5,082 for water [W], AV only [AV], AV + propionate [AV+P], and AV + CD4^{+} T depletion [AV-T] conditions, respectively) with a median cells per sample and 2,774 genes per cell. Per sample matrices were processed using the Seurat package (version 5.1.0) (5). For each sample, the miQC tool (6) was utilized to filter cells with high mitochondrial content and quality. Samples then were merged on total expression, followed by scaling to 10,000, and subsequent log normalization. The identification of 3,000 highly variable genes, considering mean expression, involved scaling the data with simultaneous regression of unwanted variation (UMI counts and the fraction of mitochondrial reads). These scaled counts served as input for PCA. Employing the IntegrateLayers function, we conducted harmony integration on the first 50 PCA components. The first 30 dimensions of the integrated data were utilized to construct a UMAP plot and for further analysis. The FindNeighbors function on integrated data was run to create an SNN (shared nearest neighbor) graph, assisting in the identification of 21 clusters by the FindClusters function with a resolution of 0.6. Markers for cell-type identification were determined using the Wilcoxon rank-sum test from the FindAllMarkers function. Genes expressed in more than 10% of cells with at least a 0.10-fold difference were considered for each cluster. Based on obtained signatures, we deleted clusters without marker genes and repeated all steps starting from data scaling. The resulting dataset, comprising 21,129 cells (5,528 + 4,908 + 6,598 + 4,095 for water, AV, AV + propionate, and AV + CD4^{+} T depletion conditions, respectively) in 16 clusters (resolution 0.6), was passed to FindMarkers function to perform differential expression testing between conditions of specific clusters. Gene set enrichment analysis (GSEA) (7, 8) was performed to identify pathways represented by sets of differentially expressed genes in different cell populations for

each condition (AV, AV + propionate, and AV + CD4$^+$ T depletion) compared to water treatment. For heatmaps, expression values were aggregated by conditions and clusters by AverageExpression function and visualized using the Phantasus app (9).

Cell-to-cell interaction modeling from single cell RNA sequencing. CellChat (version 2.1.2) (10) was employed to analyze changes in receptor-ligand interactions among joint-associated cells across different experimental conditions. Interaction networks were constructed for each condition, encompassing five cell groups: Ccr2$^+$ infiltrating monocytes/macrophages (clusters 1, 2, 3, and 4), CD4$^+$ T cells (clusters 6 and 9), CD4$^+$ T regulatory cells (cluster 5), synovial macrophages (clusters 7 and 11), and osteoclast-like cells (cluster 0). Initially, ligands and receptors for each group were identified using the identifyOverExpressedGenes and identifyOverExpressedInteractions functions. Interactions were then inferred using the computeCommunProb function with the "triMean" parameter, followed by pathway-level analysis with the computeCommunProbPathway function. After filtering out communications with fewer than 10 cells, and aggregating networks, the merged dataset contained 607 (W), 646 (AV), 684 (AV + propionate), and 481 (AV + CD4$^+$ T depletion) communication links. Differences in network-level interactions were visualized using the netVisual_diffInteraction function. Up- and down-regulated cell-cell communications were determined through a combination of identifyOverExpressedGenes and netMappingDEG.

SUPPLEMENTARY TEXT REFERENCES

1. Mack M, Cihak J, Simonis C, Luckow B, Proudfoot AE, Plachy J, et al. Expression and characterization of the chemokine receptors CCR2 and CCR5 in mice. *J Immunol.* 2001;166(7):4697-704.
2. Fox JM, Long F, Edeling MA, Lin H, van Duijl-Richter MKS, Fong RH, et al. Broadly Neutralizing Alphavirus Antibodies Bind an Epitope on E2 and Inhibit Entry and Egress. *Cell.* 2015;163(5):1095-107.

3. Koropatkin NM, Martens EC, Gordon JI, and Smith TJ. Starch catabolism by a prominent human gut symbiont is directed by the recognition of amylose helices. *Structure.* 2008;16(7):1105-15.
4. Salyers AA, Shoemaker N, Cooper A, D'Elia J, and Shipman JA. Genetic methods for Bacteroides species. *Method Microbiol.* 1999;29:229-49.
5. Hao Y, Stuart T, Kowalski MH, Choudhary S, Hoffman P, Hartman A, et al. Dictionary learning for integrative, multimodal and scalable single-cell analysis. *Nat Biotechnol.* 2024;42(2):293-304.
6. Hippen AA, Falco MM, Weber LM, Erkan EP, Zhang K, Doherty JA, et al. miQC: An adaptive probabilistic framework for quality control of single-cell RNA-sequencing data. *PLoS Comput Biol.* 2021;17(8):e1009290.
7. Subramanian A, Tamayo P, Mootha VK, Mukherjee S, Ebert BL, Gillette MA, et al. Gene set enrichment analysis: a knowledge-based approach for interpreting genome-wide expression profiles. *Proc Natl Acad Sci U S A.* 2005;102(43):15545-50.
8. Mootha VK, Lindgren CM, Eriksson KF, Subramanian A, Sihag S, Lehar J, et al. PGC-1alpha-responsive genes involved in oxidative phosphorylation are coordinately downregulated in human diabetes. *Nat Genet.* 2003;34(3):267-73.
9. Kleverov M, Zenkova D, Kamenev V, Sablina M, Artyomov MN, and Sergushichev AA. Phantasus, a web application for visual and interactive gene expression analysis. *Elife.* 2024;13.
10. Jin S, Plikus MV, and Nie Q. CellChat for systematic analysis of cell-cell communication from single-cell transcriptomics. *Nat Protoc.* 2025;20(1):180-219.

SUPPLEMENTARY FIGURES AND TABLES

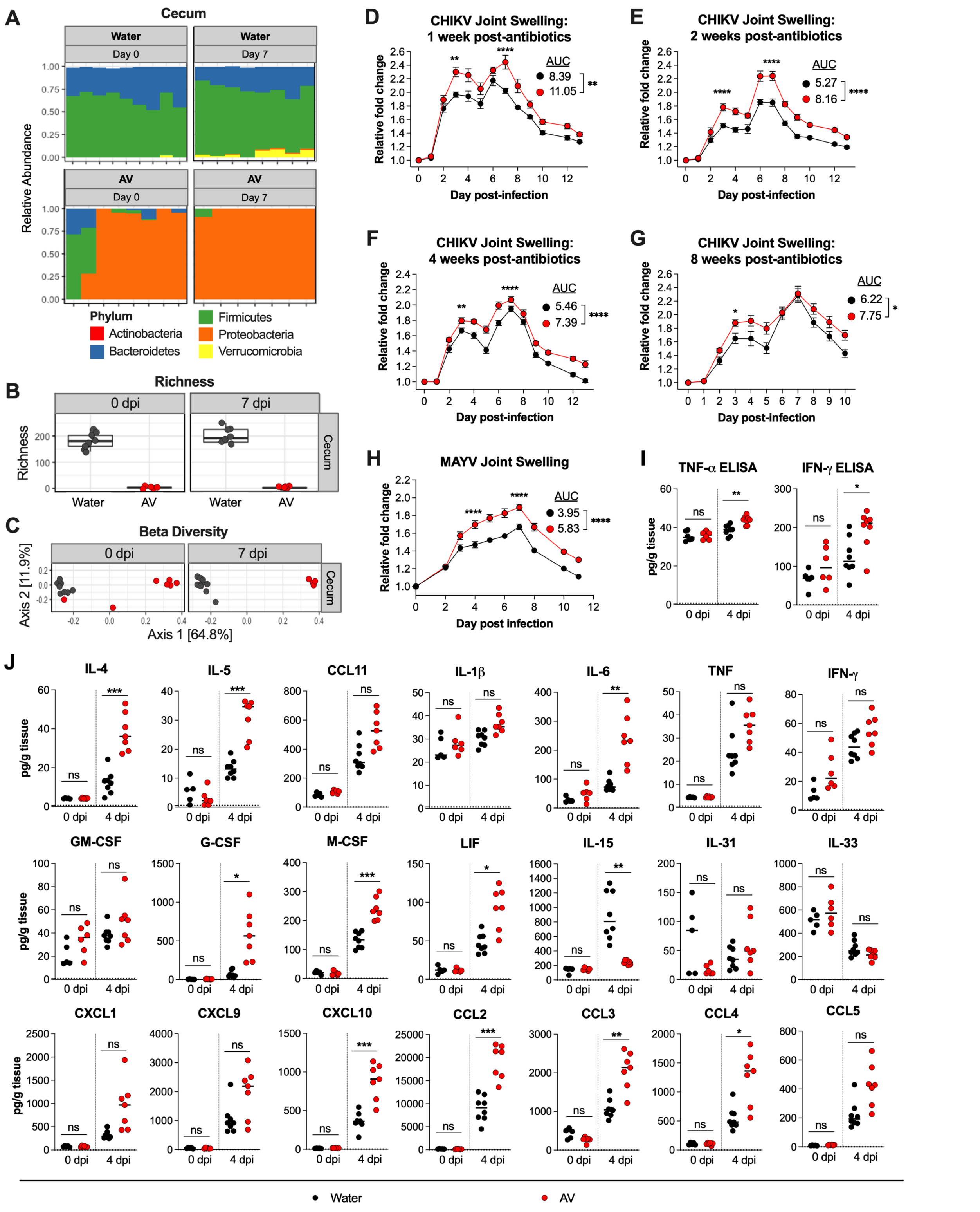

Supplemental Figure 1. Oral antibiotic treatment alters bacterial composition in cecal and colonic contents and has a sustained effect in exacerbating alphavirus-induced foot

swelling. (**A-C**) Cecal contents were collected from water- or AV-treated mice at 0 or 7 dpi. (**A**) Relative abundance of bacterial phyla detected (bacterial phyla represented in less than 1% per sample were removed), (**B**) number of bacterial taxa (richness), and (**C**) bacterial beta diversity (weighted UniFrac distance) of cecal contents (2 experiments, n = 8-9 mice per group). (**D-G**) Foot swelling in water- or AV-treated mice after CHIKV infection at (**D**) 1, (**E**) 2, (**F**) 4, or (**G**) 8 weeks post cessation of antibiotics (2 experiments, n = 7-8 mice per group). (**H**) Foot swelling in water- or AV-treated mice after MAYV infection (2 experiments, n = 9-10 per group). (**I**) TNF and IFN-γ levels in joint homogenates from water- and AV-treated mice at 0 and 4 dpi, as measured by ELISA (n = 5-8 per group). (**J**) Cytokine and chemokine levels in joint homogenates from water and AV-treated mice at 0 and 4 dpi, as measured by BioPlex assay (n = 5-8 per group). Statistical analysis: **B**, Wilcoxon tests; **C**, permutational multivariate analysis of variance (ADONIS); **D-H**, mean $\pm$ SEM, two-way ANOVA with Šídák's post-test, or unpaired t-test for analysis of AUC; **I**, unpaired t-test; **J**, unpaired t-test with Bonferroni correction for multiple comparisons. **** $P < 0.0001$; *** $P < 0.001$; ** $P < 0.01$; * $P < 0.05$; ns, not significant.

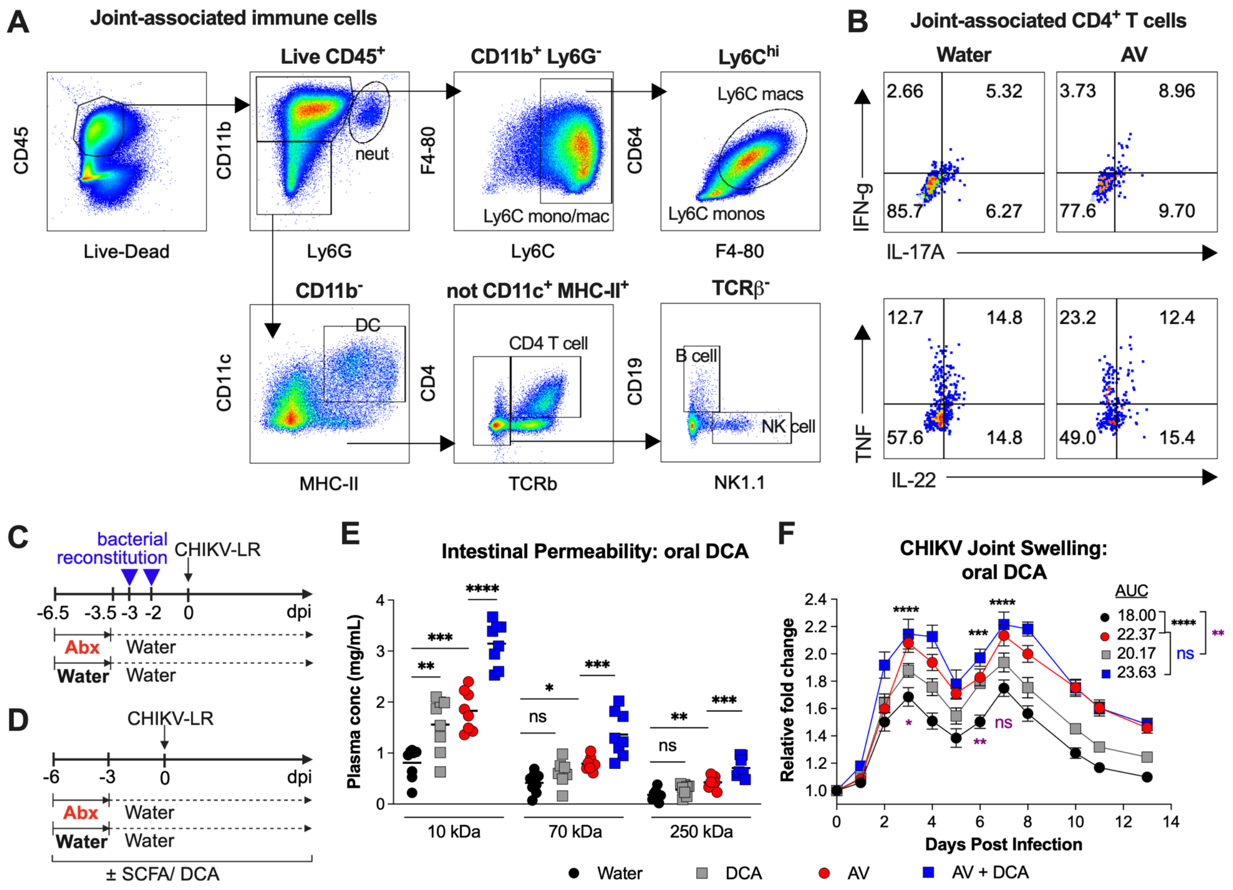

Supplemental Figure 2. Gating scheme of immune cell subsets and oral metabolite supplementation. (**A**) Gating scheme for immune cell subsets in joint-associated tissues after CHIKV infection. (**B**) Representative flow cytometry plots showing intracellular cytokine staining in $CD4^+$ T cells from joint-associated tissues of water or AV-treated mice at 4 dpi. (**C-D**) Schematics of treatments for mice that received (**C**) bacterial reconstitution or (**D**) exogenous supplementation with either individual SCFAs (butyrate, propionate, or acetate) or DCA in drinking water. (**E-F**) Mice were treated with water or AV, with or without DCA supplementation, and subsequently inoculated with CHIKV. (**E**) Intestinal permeability as measured by plasma concentrations of 10, 70, and 250 kDa dextran at 1.5 h after oral gavage (2 experiments, n = 8-9 per group). (**F**) Foot swelling after CHIKV infection in water or AV-treated mice receiving either water or SCFA supplementation with DCA (2 experiments, n = 8 per group). Statistical analysis: **E**, one-way ANOVA with Šídák's multiple comparisons test. **F**, mean ± SEM, two-way ANOVA with Šídák's post-test, or one-way ANOVA with Dunnett's multiple comparisons for analysis of AUC. **** $P < 0.0001$; *** $P < 0.001$; ** $P < 0.01$; * $P < 0.05$; ns, not significant.

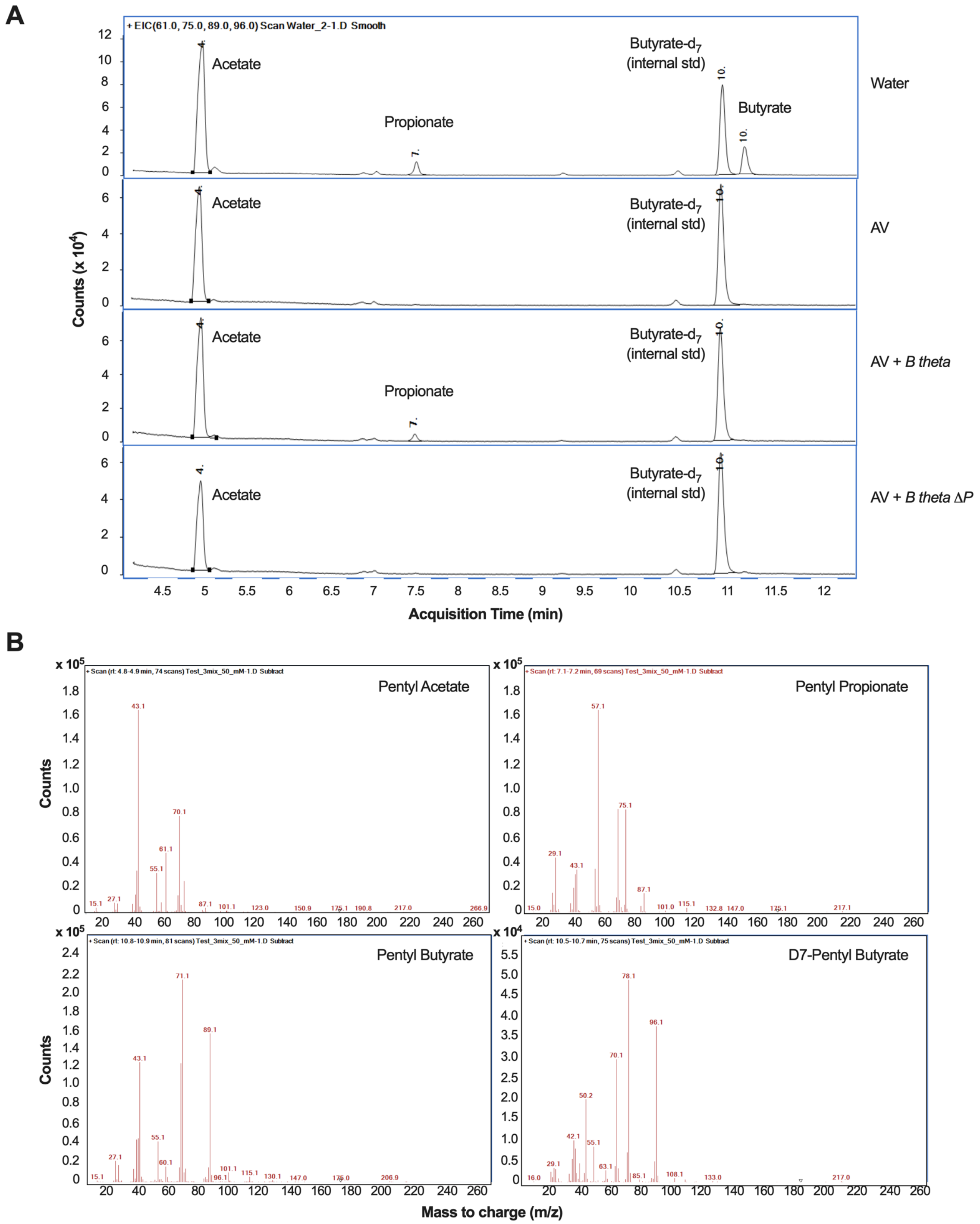

Supplemental Figure 3. Gas Chromatography-Mass Spectrometry chromatograms for evaluation of cecal SCFA. (**A**) Representative chromatograms showing intensity (counts) by retention time of processed cecal samples from control, AV-treated, and AV-treated mice colonized with either wild-type or mutant (*ΔP*) *B. thetaiotaomicron*. (**B**) Mass spectra showing intensity by the mass to charge ratio for the indicated standards.

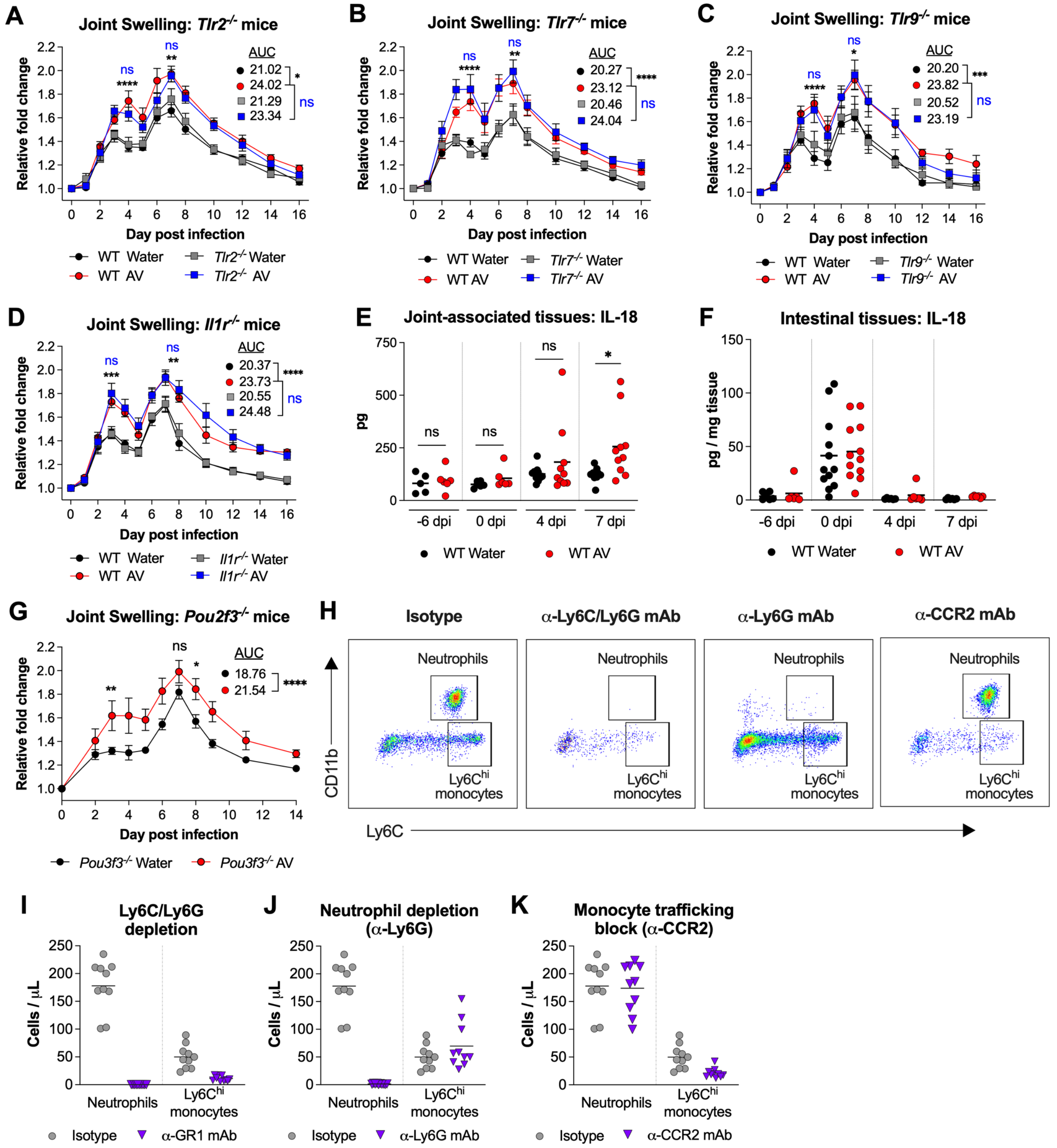

Supplemental Figure 4. Increased CHIKV-induced joint swelling after antibiotic treatment is not affected by loss of TLR2, TLR7, TLR9, or IL-1R signaling or by an absence of Tuft cells. (**A-D**) Foot swelling in water or AV-treated (**A**) *Tlr2*$^{-/-}$, (**B**) *Tlr7*$^{-/-}$, (**C**) *Tlr9*$^{-/-}$ or (**D**) *Il1r*$^{-/-}$, and WT littermate mice after CHIKV infection (2 to 5 experiments, n = 7-24 per group). (**E-F**) IL-18 cytokine levels in (**E**) joint-associated tissues or (**F**) intestinal tissues of water- or AV-treated mice at the indicated timepoints (2-3 experiments, n = 6-12 per group). (**G**) Foot swelling after CHIKV infection in water or AV-treated *Pou2f3*$^{-/-}$ mice (2 experiments, n = 6-9 per group). (**H**) Representative flow cytometry plotsof Ly6C^{hi} monocytes and neutrophils from peripheral blood of

mice treated with isotype control, anti-Ly6C/LyG, anti-Ly6G, or anti-CCR2 mAbs; , gated on CD11b^{+}. (**I-K**) Frequencies of neutrophils and Ly6C^{hi} monocytes in peripheral blood following depletion with isotype control or mAbs against (**I**) Ly6G/Ly6G (GR-1), (**J**) Ly6G, and (**K**) CCR2. Statistical analysis: **A-D, G,** mean ± SEM, two-way ANOVA with Dunnett's multiple comparisons (**A-D**) or Šídák's post-test (**G**); for analysis of AUC, one-way ANOVA with Dunnett's multiple comparisons (**A-D**) or unpaired t-test (**G**). **E,** unpaired t-test. **** $P < 0.0001$; *** $P < 0.001$; ** $P < 0.01$; * $P < 0.05$; ns, not significant.

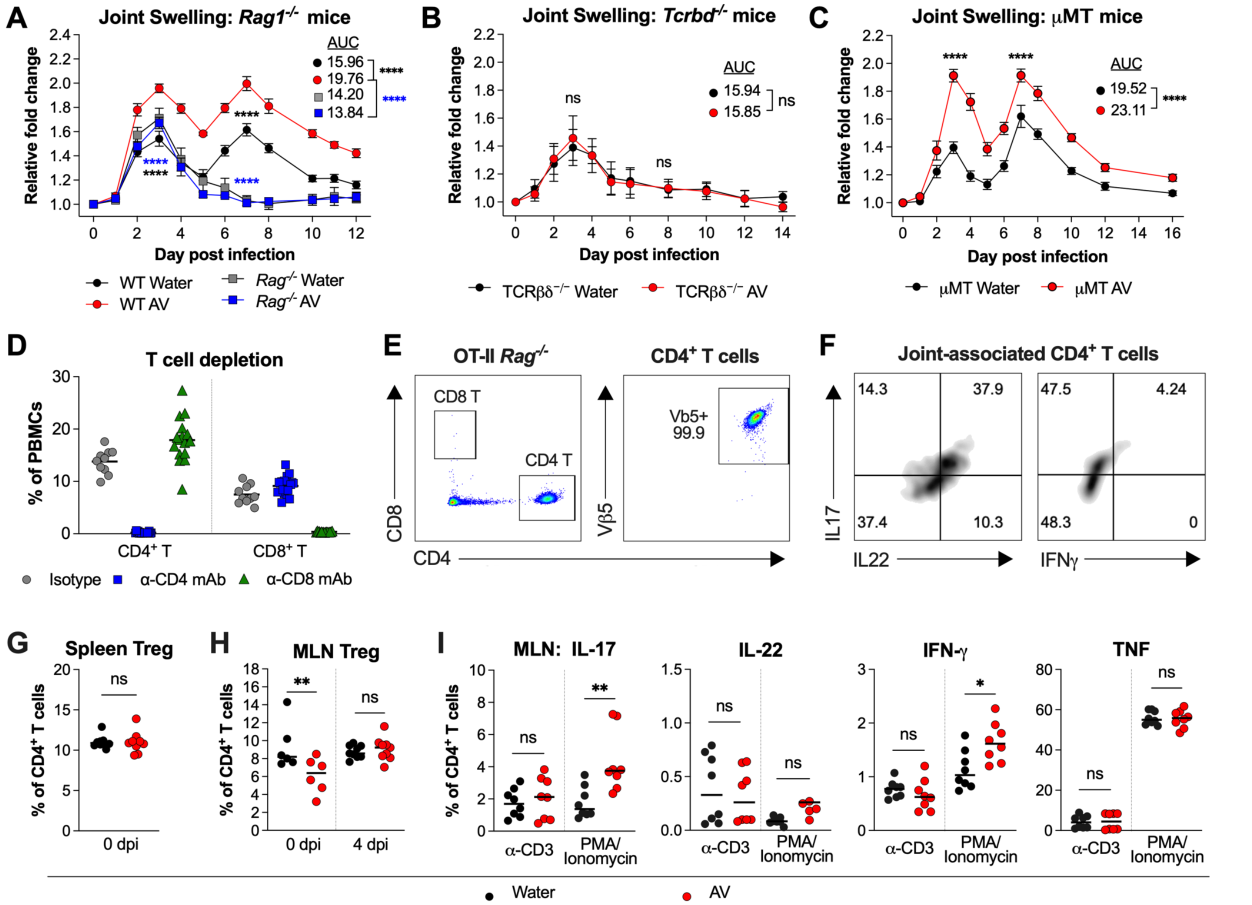

Supplemental Figure 5. Enhanced CHIKV-induced joint swelling after antibiotic treatment requires T cells. (**A-C**) Foot swelling in water- or AV-treated (**A**) *Rag*$^{-/-}$ (2 experiments, n = 9-10 per group), (**B**) *Tcrbd*$^{-/-}$ (2 experiments, n = 7-8 per group), or (**C**) μMT mice (2 experiments, n = 9 per group) after CHIKV infection. (**D**) Frequency of CD4^{+} and CD8^{+} T cells in the peripheral blood of mice following depletion with mAbs against CD4 and CD8 (2-3 experiments, n = 8-16 per group). (**E-F**) Representative flow cytometry plots showing (**E**) CD4^{+} and CD8^{+} T cells in the spleens of OT-II *Rag*$^{-/-}$ mice (virtually all CD4^{+} T cells express the transgenic, MHC-II restricted, TCR [Vα2/Vβ5] against chicken ovalbumin) and (**F**) production of the indicated cytokines by CD4^{+} T cells from the joint tissue of OT-II mice. (**G-H**) Percentages of CD25^{+} FoxP3^{+} Tregs of total CD4^{+} T cells in the (**G**) spleen and (**H**) mesenteric lymph nodes (MLN) of water- or AV-treated mice at the indicated timepoints (2 experiments, n = 6-9 per group). (**I**) Percentages of CD4^{+} T cells from the MLNs of water- or AV-treated mice that produce IL-17, IL-22, and IFN-γ in response to *ex vivo* stimulation with anti-CD3 mAb or PMA/ionomycin (2 experiments, n = 8 per group). Statistical analysis: **A-C**, mean ± SEM, two-way ANOVA with Dunnett's multiple comparisons (**A**) or Šídák's post-test (**B-C**); for analysis of AUC, one-way ANOVA with Dunnett's multiple comparisons (**A**) or unpaired t-test (**B-C**). **G-I,** unpaired t-test. **** $P < 0.0001$; *** $P < 0.001$; ** $P < 0.01$; * $P < 0.05$; ns, not significant.

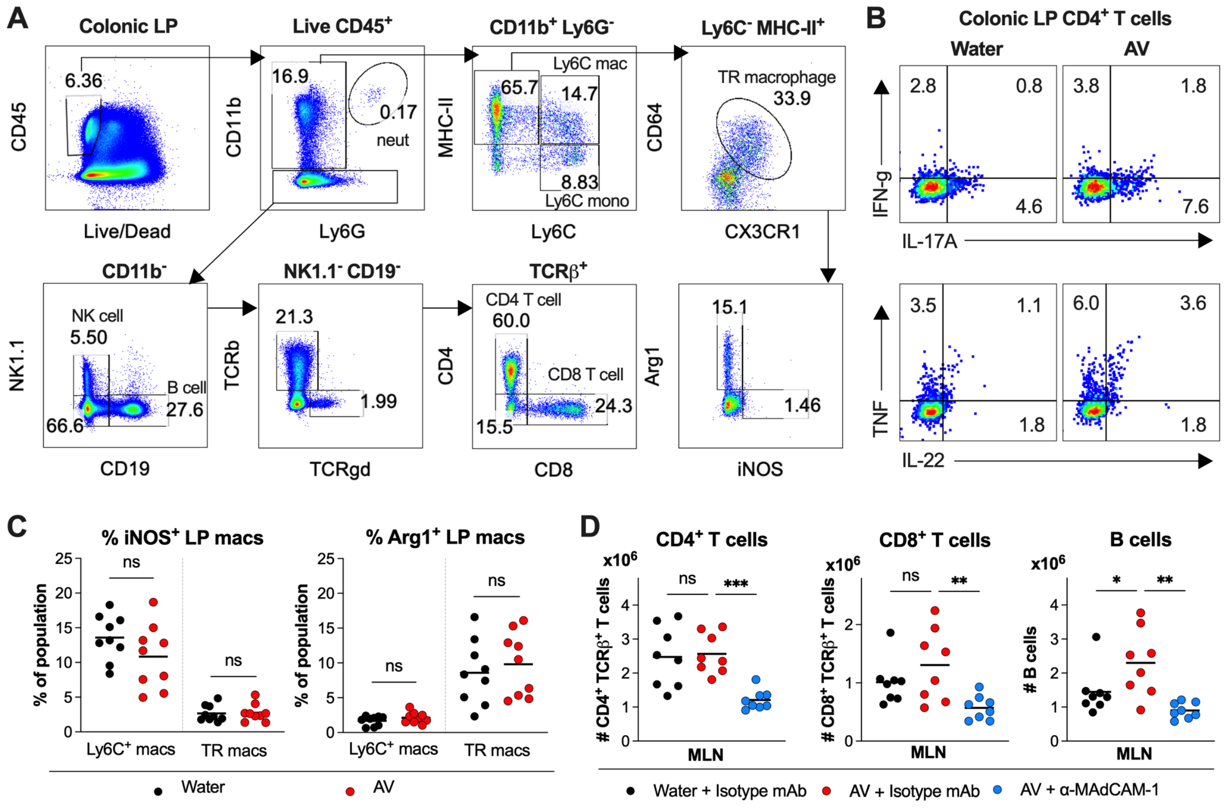

Supplemental Figure 6. Splenic, mesenteric lymph node, and lamina propria T cells in water- or antibiotic-treated mice. (**A**) Gating scheme for immune cell subsets in the colonic lamina propria. (**B**) Representative flow cytometry plots showing intracellular cytokine staining in $CD4^+$ T cells from the colonic lamina propria of water- or AV-treated mice. (**C**) Percentages of iNOS- and Arg1-expressing macrophages in the colonic lamina propria of water- or AV-treated mice. (**D**) Numbers of the indicated immune cells in the mesenteric lymph nodes of mice that received either isotype control or mAbs against MAdCAM-1 prior to treatment with water or AV and subsequent CHIKV infection (2 experiments, n = 8 per group). Statistical analysis: **C,** unpaired t-test. **D,** one-way ANOVA with Dunnett's multiple comparisons test. **** $P < 0.0001$; *** $P < 0.001$; ** $P < 0.01$; * $P < 0.05$; ns, not significant.

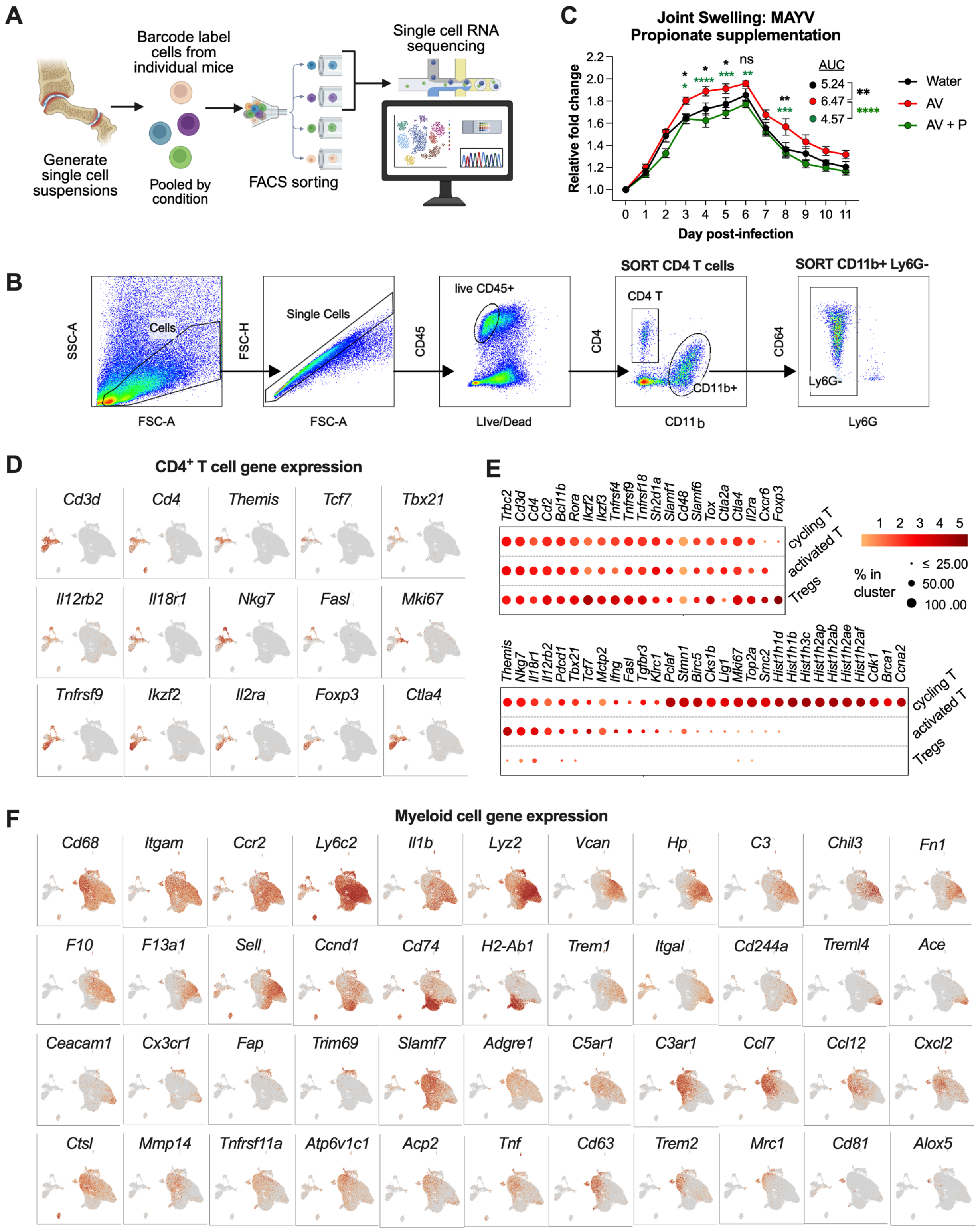

Supplemental Figure 7. Gene expression in joint-associated CD4+ T and myeloid cells. (**A**) Schematic of experimental design for single cell transcriptomic analysis of joint-associated CD4+ T cells, monocytes, and macrophages. (**B**) Gating for flow cytometry sort enrichment of joint-

associated $CD4^+$ T cells, monocytes, and macrophages for single cell RNA sequencing. (**C**) Foot swelling after MAYV infection, in WT mice treated with water, AV, or AV plus propionate supplementation (2 experiments, n = 6-7 per group). (**D**) UMAP visualization of enriched $CD4^+$ T and myeloid cells from single cell-RNA sequencing data, showing expression of the indicated genes in $CD4^+$ T cells. (**E**) Bubble plots showing patterns of gene expression amongst $CD4^+$ Tregs, activated T cells, and proliferating T cells. (**F**) UMAP visualization of enriched $CD4^+$ T and myeloid cells from single cell-RNA sequencing data showing expression of the indicated genes. Statistical analysis: **C,** mean ± SEM, two-way ANOVA with Dunnett's multiple comparisons test; for analysis of AUC, one-way ANOVA with Šídák's multiple comparisons. **** $P < 0.0001$; *** $P < 0.001$; ** $P < 0.01$; * $P < 0.05$.

Supplemental Table S1: Cytokine and chemokine concentrations in homogenates from joint-associated musculoskeletal tissues of untreated and AV-treated mice

Cytokine/ Chemokine	Concentration (mean ± SEM) (pg/g)					
	0 dpi		4 dpi		7 dpi	
	Water	AV	Water	AV	Water	AV
**IL-4**	1.4 ± 0.4	1.2 ± 0.2	3.0 ± 0.8	8.1 ± 1.2	1.5 ± 0.3	1.2 ± 0.03
**IL-6**	17.9 ± 4.4	20.6 ± 7.6	38.2 ± 7.4	66.9 ± 11.0	39.1 ± 12.1	42.9 ± 14.3
**CXCL10**	9.5 ± 2.6	9.6 ± 2.7	557.5 ± 116.0	962.2 ± 210.5	1012.5 ± 329.7	838.6 ± 197.2
**G-CSF**	0.3 ± 0.05	0.3 ± 0.01	29.5 ± 14.9	55.9 ± 16.6	8.0 ± 5.1	4.8 ± 2.6
**CCL2**	18.4 ± 9.9	10.5 ± 0.2	4830.8 ± 1164.1	9413.6 ± 1076.1	9823.0 ± 3165.4	9281.9 ± 2873.0
**CCL3**	86.6 ± 39.2	118.8 ± 36.1	840.5 ± 159.4	1379.9 ± 170.3	1163.8 ± 359.6	719.3 ± 180.1
**CCL4**	45.2 ± 8.1	52.7 ± 0.9	220.6 ± 92.5	425.5 ± 89.2	705.9 ± 235.1	470.4 ± 155.1
**CCL5**	22.3 ± 9.5	11.2 ± 5.3	259.2 ± 50.0	412.3 ± 48.3	692.1 ± 251.3	472.9 ± 125.2
**CXCL9**	98.4 ± 41.1	115.0 ± 41.9	2003.7 ± 389.8	2983.1 ± 358.8	5335.0 ± 1992.1	3327.2 ± 817.0
**CXCL1**	80.1 ± 22.2	68.6 ± 14.3	535.7 ± 149.5	641.8 ± 84.0	553.9 ± 185.5	404.7 ± 127.7
**TNF-α**	1.3 ± 0.2	1.5 ± 0.03	5.8 ± 2.7	9.4 ± 3.4	41.0 ± 15.5	43.6 ± 9.2
**IFN-γ**	5.5 ± 2.7	7.6 ± 4.8	26.9 ± 14.8	42.4 ± 14.7	205.5 ± 84.9	93.7 ± 23.3
**LIF**	12.3 ± 4.2	10.8 ± 1.9	30.5 ± 6.1	34.4 ± 3.4	25.6 ± 8.0	20.4 ± 4.9
**CCL11**	291.6 ± 94.9	285.5 ± 65.8	338.3 ± 29.6	314.2 ± 37.6	341.8 ± 74.5	261.2 ± 35.4
**IL-1α**	577.3 ± 276.9	383.7 ± 136.2	433.2 ± 298.0	256.0 ± 105.2	477.3 ± 177.0	463.0 ± 194.1
**IL-1β**	40.4 ± 9.3	44.9 ± 8.1	37.5 ± 7.8	8.2 ± 6.5	44.6 ± 10.4	28.8 ± 8.8
**IL-2**	49.2 ± 16.1	50.8 ± 21.8	12.3 ± 5.1	12.4 ± 5.7	60.0 ± 17.3	52.6 ± 19.4
**IL-9**	128.5 ± 46.4	154.2 ± 70.1	56.2 ± 10.8	51.0 ± 18.4	73.2 ± 21.3	116.8 ± 62.3
**IL-17**	1.0 ± 0.3	1.0 ± 0.2	1.2 ± 0.2	0.4 ± 0.1	0.7 ± 0.3	0.2 ± 0.05
**CXCL2**	356.0 ± 91.1	397.1 ± 115.6	196.9 ± 54.5	290.0 ± 111.4	351.6 ± 86.9	414.9 ± 134.4

Cytokine and chemokine concentrations in homogenates from joint-associated musculoskeletal tissues of untreated and AV-treated mice at 0, 4, and 7 days post-CHIKV infection. Data are presented as mean ± SEM (2 experiments, n = 6 per group).

Supplemental Table S2: Cytokine and chemokine concentrations in serum from untreated and AV-treated mice

Cytokine/ Chemokine	Concentration (mean ± SEM) (pg/mL)					
	0 dpi		4 dpi		7 dpi	
	Water	AV	Water	AV	Water	AV
**CCL11**	4004.0 ± 298.5	3709.9 ± 425.5	3933.0 ± 277.3	5122.2 ± 599.7	2325.9 ± 219.0	2854.3 ± 228.7
**CCL5**	76.9 ± 13.5	62.8 ± 2.7	109.4 ± 9.3	144.0 ± 16.3	92.6 ± 5.9	96.3 ± 7.1
**CCL2**	332.1 ± 51.7	219.9 ± 18.9	485.1 ± 73.0	577.1 ± 84.3	166.0 ± 20.0	244.0 ± 34.8
**CCL4**	17.2 ± 4.6	11.2 ± 1.2	30.0 ± 5.0	32.4 ± 5.2	8.3 ± 0.8	10.8 ± 1.8
**G-CSF**	36.0 ± 5.8	20.1 ± 4.3	73.9 ± 29.1	84.9 ± 14.3	37.2 ± 5.8	14.7 ± 2.0
**IL-12 p40**	250.5 ± 45.7	236.2 ± 54.2	272.0 ± 33.7	261.8 ± 30.1	155.6 ± 21.0	180.6 ± 32.6
**IL-12 p70**	117.2 ± 7.6	75.9 ± 8.0	133.6 ± 20.1	133.1 ± 23.4	84.3 ± 10.3	127.6 ± 23.1
**IL-4**	6.7 ± 0.7	5.3 ± 1.3	7.0 ± 1.7	7.3 ± 2.4	5.7 ± 0.4	6.6 ± 0.8
**IL-10**	104.6 ± 7.3	76.8 ± 6.7	171.2 ± 14.9	172.5 ± 15.4	77.8 ± 8.1	117.9 ± 14.4
**IL-13**	29.9 ± 8.6	10.5 ± 4.1	33.4 ± 12.1	14.5 ± 9.4	11.2 ± 4.3	18.7 ± 9.2
**CXCL1**	131.2 ± 5.4	118.0 ± 5.9	118.8 ± 8.9	109.3 ± 8.6	63.3 ± 4.2	87.6 ± 5.1
**IL-1α**	14.3 ± 1.7	13.8 ± 2.9	15.8 ± 3.0	14.0 ± 3.1	6.0 ± 1.4	10.1 ± 2.5
**IL-6**	11.2 ± 5.5	3.8 ± 0.4	12.0 ± 5.1	9.7 ± 2.2	2.7 ± 0.3	3.2 ± 0.6
**IFN-γ**	26.1 ± 5.7	14.5 ± 2.5	21.4 ± 2.8	22.2 ± 3.5	13.9 ± 2.8	18.5 ± 3.1
**IL-17**	192.2 ± 22.4	136.1 ± 21.3	189.1 ± 14.8	145.4 ± 17.1	97.7 ± 17.1	131.4 ± 25.9
**TNF-α**	242.9 ± 55.2	126.2 ± 15.3	254.1 ± 63.6	176.0 ± 24.2	103.4 ± 12.6	134.2 ± 30.8
**IL-9**	117.9 ± 41.6	53.4 ± 7.5	70.1 ± 7.3	64.6 ± 7.4	20.1 ± 3.2	16.7 ± 4.7

Cytokine and chemokine concentrations in serum from untreated and AV-treated mice at 0, 4, and 7 days post-CHIKV infection. Data are presented as mean ± SEM (2 experiments, n = 8-10 per group).

Supplemental Table S3: Primers for deleting mmdA genes. Related to Methods for generation of *B. thetaiotaomicron* mutant

	**Primer Sequence**
BT_2090_2091_F1	CGAATTCCTGCAGCCCGGGGGCATGGTGATCTGTTACCT
BT_2090_2091_R2	TCATCTTTATGCGGTCAGTG
BT_2090_2091_F3	CACTGACCGCATAAAGATGATTCGCACTACAGGTTACAGAG
BT-2090_2091_R4	GCTCTAGAACTAGTGGATCC ATCAGCGAACAGGTCCATAC
BT_2090_2091_Diag_F	AGACACGACGTTCCATATCC
BT_2090_2091_Diag_R	GTCCAGACGTTTCAGTTGGT

Supplemental Table S4: Antibodies used for flow cytometry. Related to Methods for flow cytometric analysis.

Antibody	Source	Identifier
TruStain FcX (anti-mouse CD16/32)	BioLegend	RRID:AB_1574975
FITC anti-mouse CD45	BioLegend	RRID:AB_312973
BUV395 anti-mouse CD45	BD Biosciences	RRID:AB_2651134
Alexa Fluor 700 anti-mouse/human CD11b	BioLegend	RRID:AB_493705
Brilliant Violet 605 anti-mouse/human CD11b	BioLegend	RRID:AB_2565431
eFluor 450 anti-MHC Class II (I-A/I-E)	eBioscience	RRID:AB_1272204
APC anti-mouse CD64 (FcγRI)	BioLegend	RRID:AB_11219205
PE/Dazzle 594 anti-mouse CD64 (FcγRI)	BioLegend	RRID:AB_2566559
FITC anti-mouse Ly-6G	BioLegend	RRID:AB_1236494
Brilliant Violet 785 anti-mouse Ly-6G	BioLegend	RRID:AB_2566317
APC-Cy7 anti-mouse Ly-6C	BioLegend	RRID:AB_10640120
Brilliant Violet 711 anti-mouse Ly-6C	BioLegend	RRID:AB_2562630
BV711 anti-mouse CX3CR1	BioLegend	RRID:AB_2565939
BV605 anti-mouse F4/80	BioLegend	RRID:AB_2562305
PE anti-mouse iNOS	eBioscience	RRID:AB_2572642
APC anti-human/mouse Arginase 1	eBioscience	RRID:AB_2734835
PE/Cyanine5 anti-mouse CD3ε	BioLegend	RRID:AB_312675
Brilliant Violet 421 anti-mouse CD3	BioLegend	RRID:AB_2562553
Super Bright 780 anti-mouse TCR gamma/delta	eBioscience	RRID:AB_2744919
Brilliant Violet 711 anti-mouse TCR β chain	BioLegend	RRID:AB_2629564
PE-Dazzle 594 anti-mouse CD4	BioLegend	RRID:AB_2563685
PE/Cyanine5 anti-mouse CD8a	BioLegend	RRID:AB_312749
Brilliant Violet 786 anti-mouse CD8a	BD Pharmingen	RRID:AB_2721167
FITC anti-mouse CD8a	BioLegend	RRID:AB_312745
APC/Cyanine7 anti-mouse CD19	BioLegend	RRID:AB_830707
PE anti-mouse CD19	BioLegend	RRID:AB_313643
PE-Cyanine7 anti-mouse NK1.1	BioLegend	RRID:AB_389364
APC anti-mouse IFN-γ	BioLegend	RRID:AB_315404
PE-Cyanine7 anti-mouse TNF	eBioscience	RRID:AB_11042728
FITC anti-mouse/rat IL-17A	eBioscience	RRID:AB_763581
PE anti-mouse IL-22	BioLegend	RRID:AB_2124255
APC anti-mouse perforin	BioLegend	RRID:AB_2721465
PE anti-mouse CD107a	BD Pharmingen	RRID:AB_1645247
FITC anti-mouse CD107a	BD Pharmingen	RRID:AB_10563067
Alexa Fluor 647 anti-mouse FOXP3	BioLegend	RRID:AB_1089115
Brilliant Violet 421 anti-mouse FOXP3	BioLegend	RRID:AB_2565933
Alexa Fluor 700 anti-mouse CD25	eBioscience	RRID:AB_891422
